# Detecting and Quantifying Wavelength‐Dependent Electrons Transfer in Heterostructure Catalyst via In Situ Irradiation XPS

**DOI:** 10.1002/advs.202205020

**Published:** 2022-11-14

**Authors:** Yukun Li, Li Wang, Fei Zhang, Wentao Zhang, Guosheng Shao, Peng Zhang

**Affiliations:** ^1^ State Center for International Cooperation on Designer Low‐Carbon and Environmental Materials (CDLCEM) School of Materials Science and Engineering Zhengzhou University Zhengzhou 450001 China

**Keywords:** binding energy, electrons transfer, energy induced, in situ irradiation XPS, wavelength‐dependent

## Abstract

The identity of charge transfer process at the heterogeneous interface plays an important role in improving the stability, activity, and selectivity of heterojunction catalysts. And, in situ irradiation X‐ray photoelectron spectroscopy (XPS) coupled with UV light optical fiber measurement setup is developed to monitor and observe the photoelectron transfer process between heterojunction. However, the in‐depth relationship of binding energy and irradiation light wavelength is missing based on the fact that the incident light is formed by coupling light with different wavelengths. Furthermore, a quantitative understanding of the charge transfer numbers and binding energy remains elusive. Herein, based on the g‐C_3_N_4_/SnO_2_ model catalyst, a wavelength‐dependent Boltzmann function to describe the changes of binding energy and wavelength through utilizing a continuously adjustable monochromatic light irradiation XPS technique is established. Using this method, this study further reveals that the electrons transfer number can be readily calculated forming an asymptotic model. This methodology provides a blueprint for deep understanding of the charge‐transfer rules in heterojunction and facilitates the future development of highly active advanced catalysts.

## Graphical abstract

The in situ irradiation X‐ray photoelectron spectroscopy coupled with a wavelength continuously adjustable light source is hired to monitor the wavelength‐dependent photoelectron transfer process between heterojunctions. The results indicate that this method can further observe and quantify the wavelength‐dependent electrons transfer in heterostructure.

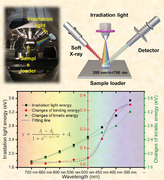

## 1 Introduction

The identity of charge transfer process at the heterogeneous interface plays an important role in improving the stability, activity and selectivity of heterojunction catalysts, thereby optimizing catalytic activity and efficiency. For the photocatalyst, the transportation pathway and reaction sites of photogenerated electrons at heterogeneous interfaces are the key to photocatalytic reaction.^[^ ^1^, ^2^, ^3^, ^4^, ^5^, ^6^ ^]^ Generally, the band structure theory companying with electron paramagnetic resonance were applied to evaluate the electrons migration through heterojunction.^[^ ^7^, ^8^, ^9^, ^10^, ^11^, ^12^, ^13^ ^]^ Traditional methods could only provide an explanation of electrons migration theoretically. However, it is a challenge to monitor and observe the photoelectron transfer process experimentally.^[^ ^14^, ^15^, ^16^, ^17^, ^18^ ^]^ Our understanding of photogenerated electrons transfer processes in heterostructure photocatalysts therefore remains limited by a lack of techniques capable of directly studying these phenomena.^[^ ^19^, ^20^, ^21^, ^22^ ^]^ In situ irradiation X‐ray photoelectron spectroscopy (ISI‐XPS) has been used to investigate the photogenerated electrons migration direction through detecting the varieties in binding energy. In our previous work, TiO_2_/Ti_3_C_2_ heterostructure was constructed to demonstrate that ISI‐XPS could be used to observe directly the migration path of photogenerated electrons.^[^ ^23^ ^]^ Besides, the method has been universally extended to more complex ternary heterojunction systems,^[^ ^24^, ^25^ ^]^ single‐atom systems,^[^ ^26^ ^]^ and dual‐single atom systems.^[^ ^27^, ^28^ ^]^

However, previous studies only focused on the change of binding energy induced by incident light, and the influence of the light wavelength was rarely mentioned. In addition, most of the irradiation light sources used in current researches are coupled by multiple wavelengths, which limits the study of changes in binding energy at specific wavelengths.^[^ ^29^, ^30^, ^31^, ^32^ ^]^ Since semiconductors behave differently in response to dissimilar wavelength light, it is essential to explore the photogenerated electrons transport behavior of semiconductors at different wavelengths. Similarly, the photocatalysts for specific conditions can be better designed through researching the binding energy changes of semiconductor at specific wavelengths. Therefore, this research can provide a deeper understanding of utilization of monochromatic light in semiconductors, so as to guide the development of photocatalyst with higher light utilization.

On the other hand, the quantitative relationship between the transfer amount of photogenerated electrons and the binding energy is ambiguous.^[^ ^33^ ^]^ Only the effect of photogenerated electrons transfer amount on binding energy has been verified but no in‐depth explanation is given.^[^ ^23^ ^]^ In addition, the existing articles have not analyzed the number of electrons transferred from the perspective of binding energy. It is of great significance to figure out the relationship between the number of photogenerated electrons and binding energy for quantifying the activity of photocatalyst. Quantitative research of electron transfer number can better combine with the existing catalyst activity characterization methods. According to the principle of ISI‐XPS, the change in binding energy can expose the migration numbers of photogenerated electrons to a certain extent owing to the binding energy was affected by the outer electron density. Consequently, a guiding method is desperately urged to establish the model of binding energy and electron transfer number. This method can extend to other more complex material systems through the study of a specific material system.

Here in this contribution, using narrow bandgap semiconductor g‐C_3_N_4_ on wide bandgap semiconductor SnO_2_ as a model heterojunction photocatalyst,^[^ ^34^, ^35^, ^36^, ^37^ ^]^ we probe the functional relationship between wavelength and binding energy for the first time. To accomplish this, ISI‐XPS technique equipped with a continuously adjustable wavelength irradiation light source is adopted to observe the photogenerated electrons behaviors at heterojunction under different wavelength light covering from visible to ultraviolet. By means of this method, the obviously change in binding energy is observed as the wavelength of the irradiation light decreases to the absorption edge of the semiconductor. For this phenomenon, a Boltzmann model is established to describe the changes of binding energy and wavelength. In addition, a new insight into correlated charge transfer in photocatalytic process is claimed by pairing the relationship between the number of transferred electrons and the binding energy. An asymptotic model was used to illustrate the change of binding energy of Sn with valence state. Based on this model, the number of electrons transferred can be roughly calculated when the binding energy changes. Our findings provide a deeper exploration of ISI‐XPS in the study of the catalytic mechanism. It is proved that this research method can be widely applied to the charge transfer between heterogeneous interfaces and carry out universal research.

## 2 Results and Discussion

### 2.1 Experimental Device and Mechanism

**Figure 1:**
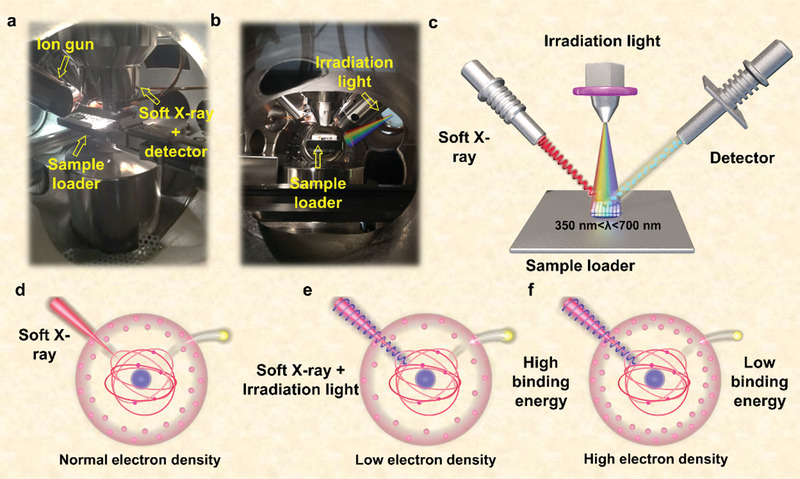
a) Front windows photographs of ISI‐XPS device. b) Side windows photographs of ISI‐XPS device. c) Schematic illustration of ISI‐XPS device. d–f) Effect of electron density on binding energy.

The equipment and mechanism we used are shown in **Figure** **1**. The photographs of ISI‐XPS equipment were given in Figure 1a,b. Figure 1c introduced the method of ISI‐XPS. Briefly, another beam of irradiation light is introduced during the XPS test process to simulate the catalytic reaction environment. The irradiation light was monochromatic light with continuously adjustable wavelength, and its range was from 700 to 350 nm, reduced by 50 nm each time. For narrow bandgap semiconductors (e.g., g‐C_3_N_4_), they could be excited under visible light. While the wide bandgap semiconductors (e.g., TiO_2_ or SnO_2_) only responded to ultraviolet light. The utilized light source in existing researches on semiconductors was mixed of variety wavelength, which hindered further exploration of monochromatic light influence. Figure 1d–f shows the mechanism of ISI‐XPS. In a typical XPS measurement, the outer electron density was usually fixed due to there were almost no free electrons (in Figure 1d). For semiconductors, however, it could be excited to produce photogenerated electrons under a suitable irradiation light. These excited electrons could migrate through the heterojunction, which result in the changes of outer electron density. As shown in Figure 1e,f, lower outer electron density will lead to the increase of binding energy, on the contrary, the binding energy will decrease.

### 2.2 Characterizations of Photocatalyst

**Figure 2:**
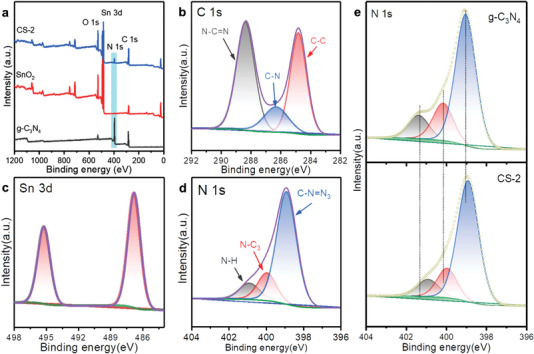
a) Wide survey of g‐C_3_N_4_, SnO_2_, and CS‐2. b) C 1s, c) Sn 3d, and d) N 1s spectra of CS‐2. e) Comparison of N 1s in g‐C_3_N_4_ and CS‐2.

Hence, a model heterostructure photocatalyst should be raised for achieving the contents discussed above. The heterojunction formed by narrow and wide band gap semiconductors could be a proper candidate for researching the photogenerated electrons under different wavelength irradiation. Thus, g‐C_3_N_4_ and SnO_2_ were chosen as the typical photocatalysts to establish the heterostructure photocatalyst model. The synthesized process was briefly shown in Figure S1 (Supporting Information). Sketchily, the calcined SnO_2_ nanotubes was placed into a sealed porcelain boat with melamine then heated to 550 °C to obtain the heterostructure photocatalyst composite. Three main peaks of SnO_2_ at (110), (101), and (211) could be clearly observed, indicating the preparation of tetragonal rutile SnO_2_ structure (Figure S2, Supporting Information). There were no other peaks existence also revealed that the SnO_2_ nanotube was without any impurities. The (002) and (100) planes could be found in pure g‐C_3_N_4_. The XRD curves of g‐C_3_N_4_/SnO_2_ composites were given above the pristine components. Because of the low loading amount and poor crystallinity, the diffraction peaks of g‐C_3_N_4_ were hardly observed in g‐C_3_N_4_/SnO_2_ composite. Thus, the Fourier transform infrared spectrometer (FTIR) was utilized to confirm the successful synthesis of g‐C_3_N_4_‐coated SnO_2_ (Figure S3, Supporting Information), which further give evidence of a Tri‐crystallinity structure. The peak around 800 cm^−1^ was derived from breathing the mode of heptazine rings. And, the multiple peaks at 1240–1700 cm^−1^ contributed to the vibration modes of CN heterocycles. Moreover, the broad peak at 3000–3400 cm^−1^ corresponding to N—H, which was associated with the unpolymerized N—H during the polymerization. Due to the low amount of g‐C_3_N_4_ in CS‐2 (the g‐C_3_N_4_ loading amount was 14 wt%, more details can be seen in the Experimental Section), the intensity was much lower than that of pure g‐C_3_N_4_. The peak at 600–750 cm^−1^ was derived from the vibration of Sn—O. Vibration peaks belonging to two components could be observed in CS‐2 indicating the successful preparation of the composite. The element distribution result of SnO_2_ showed that N did not exist on the pristine SnO_2_ nanotube (Figure S4a and Table S1, Supporting Information). While, the N, O, and Sn elements were all detected in CS‐2 (Figure S4b, Supporting Information). It was easy to notice that N distributed evenly on SnO_2_ nanotube surface meaning g‐C_3_N_4_ distributed uniformly on the surface of SnO_2_ nanotubes. The mass ratio of different elements of CS‐2 is given in Table S2 (Supporting Information). The appearance of N element in CS‐2 indicated g‐C_3_N_4_ was attached to the surface of SnO_2_ after the gas–solid reaction. The transmission electron microscope (TEM) image of SnO_2_ nanotube exhibited a tubular structure formed by the accumulation of particles (Figure S5, Supporting Information). Subsequently, the existence of g‐C_3_N_4_ was definitely demonstrated by the TEM of the heterostructure photocatalyst (Figure S6, Supporting Information). Figure S6a (Supporting Information) displayed that g‐C_3_N_4_ was attached to SnO_2_ surface in a statue of nanoparticles, which was different from the state in the previous reports.^[^ ^38^, ^39^ ^]^ This might be because SnO_2_ nanoparticles affected the polymerization process of melamine, resulting in a different state. The homogeneously distributed C, N, O, and Sn in CS‐2 was also verified through the elements mapping images (Figure S7, Supporting Information). The existence of g‐C_3_N_4_ on SnO_2_ nanotube surface was proved by both the TEM and FTIR results. The XPS measurement was hired to analyze the elemental composition and chemical state of the composite. **Figure** **2**a exhibited the wide spectra of SnO_2_ and CS samples. The binding energies were calibrated against the C 1s peak at 284.8 eV. It was easy to find the signal peaks of Sn, O, and C, meanwhile, barely N peak in pure SnO_2_ sample. In contrast, a strong characteristic peak of N 1s appeared after the solid–gas deposition reaction, indicating the successful loading of g‐C_3_N_4_. Besides, the elements intensity of Sn was a bit lower than pristine SnO_2_, mainly because the covered g‐C_3_N_4_ could weaken the signal of Sn. The narrow spectra of C, N, and Sn in CS were shown in Figure 2b–d. Consistent with other researches, the C 1s pattern displayed three components corresponding to C—C, C—N, and N—C=N, respectively.^[^ ^40^, ^41^ ^]^ The C—N and N—C=N bonds were mainly attributed to the g‐C_3_N_4_ in CS‐2. The Sn 3d_5/2_ and Sn 3d_3/2_ in CS‐2 are shown in Figure 2c. As shown in Figure 2d, the components peak of N 1s was the bonding of C—N=N, N—C_3_, and N—H, respectively. The N element mainly came from g‐C_3_N_4_; thus, the components consisted of the pristine g‐C_3_N_4_. N 1s peaks of CS‐2 and g‐C_3_N_4_ were put together for comparison to further demonstrate the existence of g‐C_3_N_4_ on the surface of SnO_2_ nanotube (Figure 2e). The chemical state of N 1s in CS‐2 was similar to that of g‐C_3_N_4_, indicating that g‐C_3_N_4_ was successfully deposited on the surface of SnO_2_ by gas–solid reaction. In addition, a slight shift of binding energy revealing the heterojunction was formed between SnO_2_ and g‐C_3_N_4_. Furthermore, comparing the Sn 3d spectra of SnO_2_ with CS‐2, it could be clearly noticed that the binding energy had a positive shift (Figure S8, Supporting Information). The opposite movement of Sn and N indicates the formation of heterojunction between the two materials.

### 2.3 ISI‐XPS Measurement of Photocatalyst

**Figure 3:**
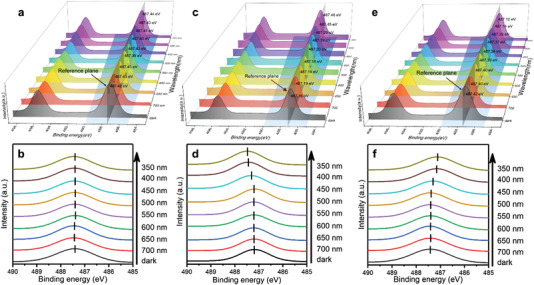
a,b) ISI‐XPS of CS‐2 before charge compensation. c,d) ISI‐XPS of SnO_2_. e,f) ISI‐XPS of CS‐2 after charge compensation. b,d,f) Enlarged view of colored square in (a), (c), and (e).

Afterward, the photogenerated electron transfer between heterogeneous interfaces was studied via the XPS equipped with a monochromatic light source with continuously adjustable wavelength. **Figure** **3**a showed the shift of binding energy of Sn 3d in CS‐2. It was surprisingly to find that Sn peaks hardly moved. In a typical XPS measurement, the samples were attached on the carbon conductive tape. The carbon conductive tape could act as an electron trap in the ISI‐XPS test for single component semiconductor, which meant the photogenerated electrons would flow from photocatalyst to carbon conductive tape and finally to the sample loader. Thus, we suspect that the electrons poured into SnO_2_ and the electrons flowing from it to the substrate had reached a balance, resulting in no significant change in the outer electron density (Figure S9a,b, Supporting Information). Based on these conditions, the binding energy change in composite would be inaccurate due to the loss of electrons which were affected by the carbon conductive tape substrate. For the pristine SnO_2_, the photogenerated electrons would flow to the substrate companying a decrease in outer electrons (Figure S9c, Supporting Information). Therefore, through the binding energy changes of SnO_2_ under light irradiation, the influence of photogenerated electrons transferred to the substrate in the heterostructure photocatalyst could be weakened by charge compensation (Figure S9d, Supporting Information).^[^ ^42^, ^43^, ^44^ ^]^ The changes of binding energy in pure SnO_2_ under different wavelength light irradiation were given in Figure 3c. As shown in Figure 3d, an enlarged view of the colored quadrilateral area in Figure 3c, the Sn 3d_5/2_ were barely moved until the wavelength was decreased to 450 nm. The positive shift of analytical peaks indicating the photogenerated electrons on SnO_2_ migrated to carbon conductive tape substrate. The binding energy changes after compensation was displayed in Figure 3e,f (all the binding energy data were given in Table S3, Supporting Information). It can be found that the shift of binding energy got more obvious, especially the light with a lower wavelength. The Sn 3d_5/2_ under 350 nm light irradiation was moved negatively about 0.30 eV compared with the dark situation. This phenomenon demonstrated that the wavelength of irradiation light could directly affect the migration of photogenerated electrons which was reflected through the change of binding energy.

**Figure 4:**
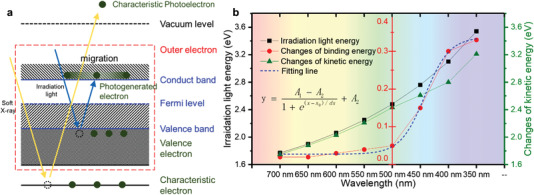
a) Different activated electrons in ISI‐XPS. b) Relationship of irradiation light energy, changes of binding energy, and kinetic energy of Sn 3d_5/2_ in CS‐2.

As was known to all, XPS technique was achieved through the photoelectric effect equation [given in Equation (1), where *hv*, *E*_k_, and *E*_b_ represented the energy of Al K*α*, kinetic energy, and binding energy). Because the excitation source energy was stationary in general tests, the binding energy of an element in a specific compound generally did not change in a single test. However, this situation would be different when the outer electron density altered. The wavelength of irradiation light was usually 300–700 nm in ISI‐XPS measurement, which is enough to excite semiconductors to produce photogenerated electrons though its energy was much smaller than that of X‐ray excitation source. In **Figure** **4**a, for heterostructure photocatalyst, electrons in the valence band would be excited by suitable wavelength light and migrate to participate in photocatalytic reaction. The relationship between irradiation light energy, changes of binding energy, and kinetic energy was shown in Figure 4b. It could be seen that the binding energy hardly changes until the wavelength was reduced to 500 nm. This phenomenon occured because the heterojunction photocatalyst could not be excited to produce free photogenerated electrons under irradiation light with low energy. But the outer valence electrons still absorbed this energy and increased the activity of outer electrons, which was why the kinetic energy would change. In this condition, the kinetic energy under light irrdiation was a bit larger than the dark one, which was defined as *E*_k_′. When the wavelength of the irradiated light is reduced to 450 nm, photogenerated electrons were generated and transferred at the heterogeneous interface. Migration of photogenerated electrons would lead to the loss of electrons and the change of electron density. In this situation, the increase in kinetic energy would no longer be approximately equal to the energy of the incident light. Herein, this kinetic energy under this condition was defined as *E_k_* ″. Owing to lack of reactant, the transferred photogenerated electrons could not be consumed through the catalytic reactions, resulting in aggregation and increased electron density and finally affected the binding energy [given in Equation (2), where *E*_IL_, Δ*E*_b_, and *E*_g_ are represented for incident light energy, changes in binding energy, and bandgap, respectively). The blue dashed line in Figure 4b was the fitting function of wavelength and changes of binding energy. In the fitting process, the horizontal and vertical coordinates only consider the value. The formula of Boltzmann function was given as inset figure. Equation (3) was the fitting function, where *x* and *y* represented the wavelength and changes of binding energy, respectively

(1) $$hv=E_{k}+E_{b}$$

(2) $$\mathrm{hv}+E_{\mathrm{IL}}=\left\{\begin{array}{cc} E_{k}^{\prime }+E_{b}, & E_{\mathrm{IL}}<E_{g}\\ E_{k}^{\prime \prime }+E_{b}+\Delta E_{b}, & E_{\mathrm{IL}}>E_{g} \end{array}\right.$$

(3) $$y=\frac{0.31}{1+e^{\left(x-411.54\right)/dx}}+0.027$$

**Figure 5:**
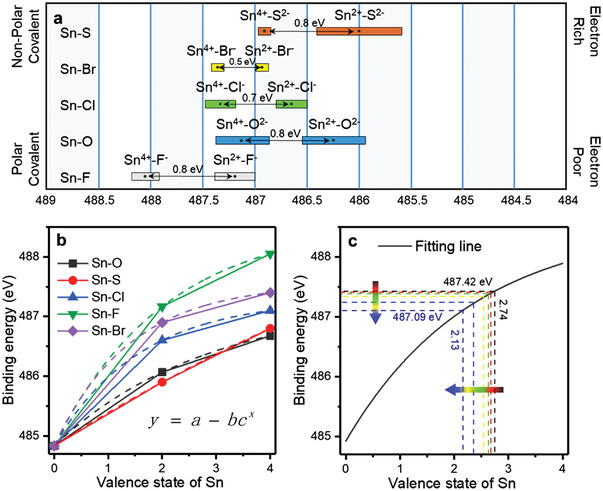
a) Relationship of binding energy and valence state in different Sn compounds.^[^ ^45^, ^46^, ^47^, ^48^, ^49^, ^50^, ^51^ ^]^ b) Binding energy and valence state of Sn in different compounds. c) Fitting curve of Sn in CS‐2.

Here, it is well known that the binding energy is closely relative to the chemical environment of a certain atom. Changes in binding energy for this reason are called chemical shift. In the past few decades, many physicists had studied the relationship between the changes of binding energy and chemical shift and concluded that the ratio of binding energy changes and chemical shift was a specific constant, which was quite different in various elements. According to the published articles, a brief summary of the binding energy of Sn 3d_5/2_ in several compounds was given in **Figure** **5**a. Rectangles of different colors represented the possible range of binding energy. Here, the value at the midpoint was taken as the references of binding energy analysis. Because of the varieties in the electronegativity of Sn bound anions, different compounds of Sn also showed various binding energy differences. It was an obvious result from the figure that the binding energy changed significantly when Sn^2+^ became Sn^4+^. The relationship between binding energy and valence state of Sn was shown in Figure 5b. We found that the valence state and binding energy of Sn conformed to the Asymptotic model, and the formula was given inset of Figure 5b. The parameters of each fitting curve in Figure 5b were given in Table S4 (Supporting Information). Figure 5c was the average fitting curve obtained from Figure 5b, which can roughly reflect the relationship between the binding energy and valence state of Sn in heterojunction photocatalyst CS‐2. The function relationship of binding energy and valence state was given in Equation (4), where *x* and *y* represent valence state and binding energy of Sn, respectively. Because the formation of heterojunction also affected the charge distribution, it was worth noting that the binding energy of Sn in CS‐2 is higher than that of SnO_2_ in Figure 5b. According to the results of ISI‐XPS, as the wavelength of the irradiated light gradually decreases to 350 nm, the binding energy decreases by 0.3 eV. Combined with the function in Figure 5c, the valence state of Sn decreased from 2.74 to 2.13. It can be inferred that 0.61 electrons had been transferred under 350 nm light irradiation. Changes in binding energy and electron transfer numbers at other wavelengths are given in Table S5 (Supporting Information). The trend of electron number change was similar to that of binding energy, which increased gradually with the decrease of wavelength. The above two findings gave some in‐depth understanding of the relationship between binding energy, wavelength, and electron transfer number

(4) y=488.55−3.628∗0.652∧x

### 2.4 Photocatalytic Performances Characterizations

**Figure 6:**
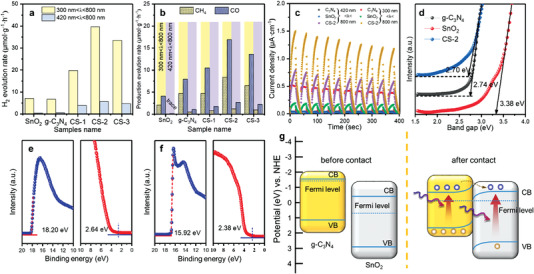
a) Photocatalytic water splitting rate and b) solar‐fuel production rate of SnO_2_, g‐C_3_N_4_, and g‐C_3_N_4_/SnO_2_ composite. c) Photocurrent response curves of g‐C_3_N_4_, SnO_2_, and CS‐2. d) Calculated bandgaps of g‐C_3_N_4_, SnO_2_, and CS‐2. UPS measurement of e) g‐C_3_N_4_ and f) SnO_2_. g) Band structure of g‐C_3_N_4_ and SnO_2_ before and after the heterojunction formed.

The foregoing discussion of ISI‐XPS investigation found that light with different wavelengths had a great impact on the properties of photocatalyst. The next step was to inspect that how different wavelengths of light affected photocatalytic and photochemical performance. The solar fuel production rate of prepared samples was shown in **Figure** **6**a,b. As expected, the hybrid samples presented a much higher production evolution rate compared with the two pristine components. Moreover, the ultraviolet light directly and drastically affected the activity of heterostructure photocatalysts. It was obvious that the activity in visible‐light range was significantly lower than that in solar‐light range. This result was consistent with the above ISI‐XPS results, that is, the strong absorption in the UV region brought more electrons to transfer. Besides, the CS‐2 showed the highest photocatalytic activity among the three composite materials. Presumably because less g‐C_3_N_4_ was not enough to produce enough photogenerated charges to participate in the reaction, while excess g‐C_3_N_4_ would cover SnO_2_ nanotube reducing the reaction sites for water splitting or CO_2_ reduction. Subsequently, the cycling test was carried out to procedure the stability of prepared heterostructure photocatalyst. The amount of H_2_ continuously raised under solar light irradiation and no apparent deactivation was observed, revealing outstanding hydrogen evolution cycling stability for long‐term photocatalytic applications (Figure S10a, Supporting Information). As for the CO_2_ reduction stability test, after 600 min the CS‐2 maintained high performance with almost no decrease (Figure S10b, Supporting Information). After the cycle reaction, the phase and morphology of the photocatalyst after the reaction was further characterized. The phase of CS‐2 did not change extensively after the reaction (Figure S11, Supporting Information). In addition, the scanning electron microscopy (SEM) images also gave a demonstration that the nanotube morphology did not be destroyed after the photocatalytic reaction (Figure S12, Supporting Information). As was known to all, the apparent quantum efficiency (AQE) was a very effective experimental method to quiz the utilization of photocatalyst to light of different wavelengths. It was also an important data to verify the ISI‐XPS test results mentioned above. As shown in Figure S13 (Supporting Information), the AQE and absorption curves of CS‐2 showed a similar trend. This result was also consistent with the conclusion of ISI‐XPS. Transient photocurrent response was another test that could directly reflect the problem of electron migration, which was shown in Figure 6c. A fast separation of photogenerated electrons and holes would lead to a high photocurrent density. The photocurrent density under solar light of all samples enhanced than that of visible light, which meant a stronger photon energy could bring more amount of photogenerated charges. The electrochemical impedance spectroscopy (EIS) spectra showed that the resistance of charge carriers transferring of composite significantly decreased owing to the formation of heterojunction (Figure S14a, Supporting Information). The CS‐2 composite exhibited the smallest radius from the Nyquist plots, revealing the electrons could migrate easier than two pristine components. A fast charge transfer contributed to the separation of photogenerated electron–hole pairs, which could lead to a higher photocatalytic activity. In addition to the above tests, linear sweep voltammetry (LSV) curves could describe the difficulty of hydrogen evolution reaction, which were given in Figure S14b (Supporting Information). Typically, the earlier the inflection point of the LSV curve appears, the lower the overpotential required for the catalytic reaction. As expected, the Tafel slope of CS‐2 (201 mV dec^−1^) was smaller than SnO_2_ (229 mV dec^−1^) and g‐C_3_N_4_ (211 mV dec^−1^), revealing that the sluggish hydrogen evolution kinetics had been significantly accelerated after the heterojunction formed (Figure S14c, Supporting Information).^[^ ^52^, ^53^, ^54^ ^]^ The photoelectrochemical performances of other composite samples were also compared to illustrate the effect of g‐C_3_N_4_ content. The CS‐2 showed the highest current density among three composites. Besides, the smallest Nyquist radius of CS‐2 indicated a more rapidly charge migration rate, which also reflected in the LSV curves. Likewise, the calculated Tafel plots revealed CS‐2 had the fastest hydrogen evolution kinetics (Figure S15, Supporting Information). The above results of three composite samples implied the proper amount of g‐C_3_N_4_ is also important for the catalyzed reaction. Additionally, the influence of specific area was investigated. Pristine g‐C_3_N_4_ was less likely to the photocatalytic reaction due to the low specific area of bulk material. The composite material used the structure of nanotubes to load g‐C_3_N_4_ on the surface of the SnO_2_ nanoparticles, which significantly increased the reaction area and made the photocatalytic reaction easier to occur. N_2_ adsorption–desorption isotherms and pore size distributions of g‐C_3_N_4_ and CS‐2 were shown in Figure S16 (Supporting Information). The Brunauer–Emmett–Teller specific area values of CS‐2 could attain ≈25.03 m^2^ g^−1^, nearly three times higher than that of pure g‐C_3_N_4_ (8.88 m^2^ g^−1^). Finally, the band structure was analyzed to more brightly describe the path of electron migration.^[^ ^55^, ^56^, ^57^, ^58^, ^59^, ^60^, ^61^, ^62^, ^63^, ^64^ ^]^ The work function (*W*_f_) of semiconductors was an important parameter to predict the electrons transfer within semiconductors heterostructure. What's more, the investigation of band structure was a useful method to figure out the electrons transfer through the heterojunction. Therefore, the UV–vis spectrum and UPS were hired to characterize the Fermi level, conduct band (CB), and valance band (VB) position of g‐C_3_N_4_ and SnO_2_. Figure S17 (Supporting Information) showed the UV–vis DRS measurement of prepared samples. The widened of the light response range and increased of light absorption indicating the formation of heterojunction. The bandgaps were calculated as 3.38 eV (SnO_2_), 2.74 eV (g‐C_3_N_4_), and 2.70 eV (CS‐2), respectively. The narrowing of bandgap demonstrates that g‐C_3_N_4_ successfully adhered to the surface of the SnO_2_ nanotube forming a heterojunction. Furthermore, the Fermi level and valance band position were measured via UPS, which was shown in Figure 6e,f. The Fermi level, conductive band, and valence band could be calculated through the equations below

(4) $$W_{f}=\mathrm{hv}-E_{\mathrm{cut}\;\mathrm{off}}$$

(5) $$\mathrm{Fermi}\;\mathrm{level}=E_{\mathrm{vac}}-W_{f}$$

(6) $$E_{\mathrm{VB}}=\mathrm{Fermi}\;\mathrm{level}+E_{\mathrm{Fermi}\;\mathrm{edge}}$$

(7) $$E_{\mathrm{CB}}=E_{\mathrm{VB}}-E_{\mathrm{bandgap}}$$

The *E*_cut off_ of g‐C_3_N_4_ and SnO_2_ was 18.20 and 15.92 eV, respectively. The *W*_f_ of g‐C_3_N_4_ and SnO_2_ were calculated as 3.02 and 5.30 eV via Equation (4), respectively. It can be read that VB was 2.64 eV lower than Fermi level in g‐C_3_N_4_ from the UPS measurement. As in SnO_2_, this value was 2.38 eV. According to Equations (6) and (7), the CB and VB position could also be obtained. The results of Mott–Schottky (M–S) showed that the conduction band positions of SnO_2_ and g‐C_3_N_4_ are −0.5 and −1.5 V versus standard hydrogen electrode (NHE), respectively (Figure S18, Supporting Information). We also compared some recent researches with similar photocatalytic system. The band position of our work and other literatures was given in Table S6 (Supporting Information). Compared with the existing publications, it was found that this photocatalytic system prepared by us had a higher conduction band position, indicating that the reduction ability of electrons was stronger. The band structure of g‐C_3_N_4_ and SnO_2_ was displayed in Figure 6g. Owing to the difference of *W*_f_, a heterojunction would form at the interface of g‐C_3_N_4_ and SnO_2_. As the two semiconductors contacted, the electrons would flow from g‐C_3_N_4_ to SnO_2_ causing the Fermi level move to the same position. At the same time, the shift of Fermi level would lead to band bending, which was embodied in the upward bending of the energy band of g‐C_3_N_4_ and the downward bending of SnO_2_. As the composite was under light irradiation, both two components would be excited to produce photogenerated electrons and holes. The photogenerated electrons migrated from g‐C_3_N_4_ to SnO_2_ through the heterojunction, which matched well with the results of ISI‐XPS. The upward bending band could prevent the photogenerated from flowing back to g‐C_3_N_4_. When the energy of irradiated light gradually increased to excite SnO_2_ to produce photogenerated electrons, the electrons on its conduction band would further increase, which showed that the binding energy moved further to the direction of negative position.

## 3 Conclusion

In summary, we establish a wavelength‐dependent Boltzmann function based on the g‐C_3_N_4_/SnO_2_ model catalyst to describe the changes of binding energy and wavelength through utilizing a continuously adjustable monochromatic light irradiation XPS technique. Besides, by analogizing the relationship between the binding energy and valence state of several Sn compounds, we also gave a functional model that can roughly calculate the electron transfer number. These results revealed that the number of photogenerated electron transfer was highly correlated with the wavelength of incident light. This work provides a strong support for deep understanding of the charge‐transfer mechanism in heterojunction and facilitates the future development of highly active advanced catalysts.

## 4 Experimental Section

### Preparation of SnO_2_ Nanotube

First, SnCl_2_·2H_2_O (0.45 g) was dispersed into ethanol (5.6 mL) and DMF (4.6 mL) solution and stirred. After the SnCl_2_·2H_2_O was resolved, PVP (1.2 g) was added slowly into the solution and stirred for 12 h. Then the obtained precursor's solution was drawn into the injector. The distance between the needle tip and collector plane was about 15 cm. The positive and negative voltage was set at 12 and −3 kV, respectively. At last, the as‐prepared precursor was calcined at 600 °C in air atmosphere for 60 min at a rate of 2 °C min^−1^ to obtain SnO_2_ nanotube.

### Preparation of g‐C_3_N_4_/SnO_2_ Composite

The composite was synthesized via a facial gas–solid reaction approach. It was fabricated as follows. Different mass of melamine was put into a corundum crucible and covered with a porous aluminum foil. Then SnO_2_ (50 mg) was evenly placed on the porous aluminum foil. After that, it was put in a tube furnace in a nitrogen atmosphere at 550 °C for 2 h with a rate of 5 °C min^−1^. After cooling down to room temperature a light‐yellow hybrid was obtained. The composite materials with different g‐C_3_N_4_ loading amounts (7, 14, and 21 wt%) obtained by adding diverse amounts of melamine were named CS‐1, CS‐2, and CS‐3, respectively.

### Materials Characterization

The field emission SEM (FESEM, JSM‐7500F, JEOL) was used to investigate the morphologies of the prepared samples. And the morphologies and structure were also studied through TEM (FEI Tecnai G2 F20). XRD (Empyrean, PANalytical B.V., Holland) was applied to study the crystallinity under a scanning rate of 4° min^−1^ from 10° to 80°. The XPS (AXIS SUPRA UltraDLD, Kratos Analytical Inc.) was used to investigate the photogenerated electrons migration pathway across the interface of heterostructure photocatalyst. UV–vis spectrophotometer (Shimadzu, model UV 3600) was taken for characterization of all samples. N_2_ adsorption–desorption curves were detected by a Micromeritics ASAP 2460 instrument. The FTIR was tested on TENSOR II, Bruker.

### ISI‐XPS Measurement

The ISI‐XPS was carried out on AXIS SUPRA UltraDLD. The continuous tunable wavelength light source was PLS‐EM 150 (Beijing Perfectlight Co. Ltd.). In a typical test, the dark environment binding energy was characterized with all lights turned off in the SAC chamber. Then the irradiation light source was turned on, the wavelength was set to 700 nm, and the test was conducted. After one round of test, the wavelength was reduced by 50 nm and the test continued. The test was finished until the test wavelength was reduced to 350 nm.

### Photocatalytic Activity Performance

Photocatalytic solar fuel produce rate was measured by gas chromatography (GC‐2014, Shimadazu). The photocatalytic hydrogen evolution reaction was carried out in a quartz reactor. And 300 W Xe lamp (MC‐PF800B, Beijing Merry Change Technology Co., Ltd.) was selected as a visible light and solar light source. Generally, 20 mg sample, 50 mL deionized water, and 10 mL triethanolamine (anhydrous, Sinopharm Chemical Regent, 99.5%) were added into the quartz reactor with stirring. The 300 W Xe lamp was used to illuminate the quartz reactor after purging with Ar. For the CO_2_ photocatalysis reduction, 10 mg sample, 10 mL deionized water, and 10 mL triethanolamine were added into the quartz reactor with stirring. Before reaction, high‐purity CO_2_ was introduced to replace the gas in the quartz reactor. The gas chromatography was equipped with a thermal conductivity detector (TCD) and flame ionization detector (FID) to detect H_2_ and CO and CH_4_ which generated from quartz reactor via liquid suspension reaction, respectively.

### Electrochemical Measurement

Electrochemical performance was characterized through an electrochemical workstation (CHI‐760E, CH Instruments, Shanghai, China) in a cubic quartz reactor. The 1 m Na_2_SO_4_ aqueous solution acted as the electrolyte. The Pt tablet served as a counter electrode and the Ag/AgCl electrode acted a reference electrode. The F‐doped tin oxide (FTO) glass coated with photocatalyst sample was the working electrode. The mixture included deionized water (225 µL), methanol (75 µL), and nafion solution (30 µL). Then 100 µL of the prepared solution was taken and dropped on the area of 1 × 1 cm^2^ of FTO glass. After it was naturally dried to form a dense film, electrochemical test was conducted. A 300 W Xe lamp (MC‐PF800B, Beijing Merry Change Technology Co., Ltd.) served as the light source. The EIS was measured from 1 MHz to 0.1 Hz and the amplitude was 50. The LSV was measured from 1 to −2 V and the sweep rate was 0.05 mV s^−1^. The M–S curve was measured from 2 to −2 V and the frequency was 1000 Hz.

## Conflict of Interest

The authors declare no conflict of interest.

## Supporting information

Supporting Information

Supporting Information

## Data Availability

The data that support the findings of this study are available from the corresponding author upon reasonable request.
