# Supplementary material for: Detecting and Quantifying Wavelength‐Dependent Electrons Transfer in Heterostructure Catalyst via In Situ Irradiation XPS
Source: Adv Sci (Weinh). 2022 Nov 14;10(4):2205020. doi: 10.1002/advs.202205020 (PMC9896054; doi:10.1002/advs.202205020)
Supplement: Supplementary file 1 — Supporting Information [file ADVS-10-2205020-s002.pdf]

Supporting Information

Detecting and Quantifying Wavelength-Dependent Electrons Transfer in Heterostructure Catalyst via In-situ Irradiation XPS

*Yukun Li, Li Wang, Fei Zhang, Wentao Zhang, Guosheng Shao, Peng Zhang**

Y. Li, L. Wang, F. Zhang, W. Zhang, Prof. G. Shao, Prof. P. Zhang

State Center for International Cooperation on Designer Low-carbon & Environmental Materials (CDLCEM), School of Materials Science and Engineering, Zhengzhou University, Zhengzhou 450001, China

E-mail: zhangp@zzu.edu.cn

Figures

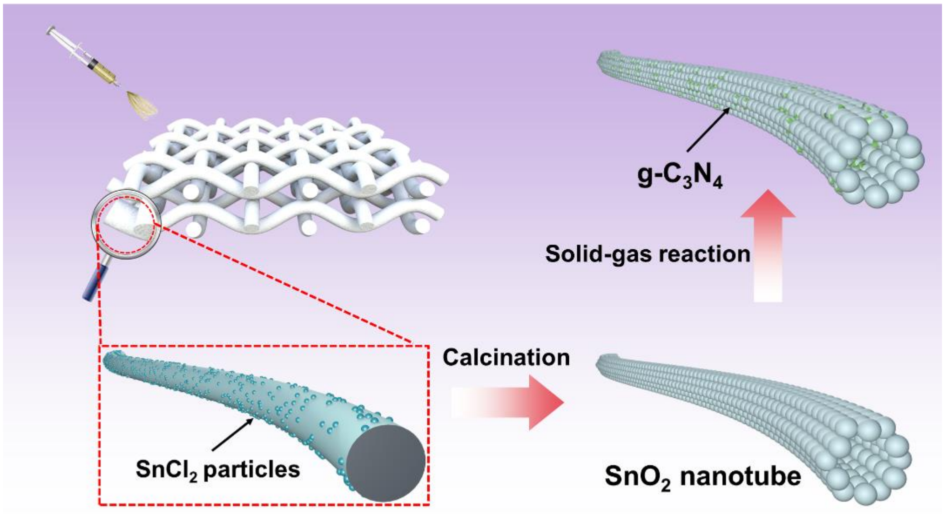

Figure S1. The Schematic illustration of the synthesis of the C_3N_4/SnO_2 nanotubes via electrospinning and gas-solid reaction method.

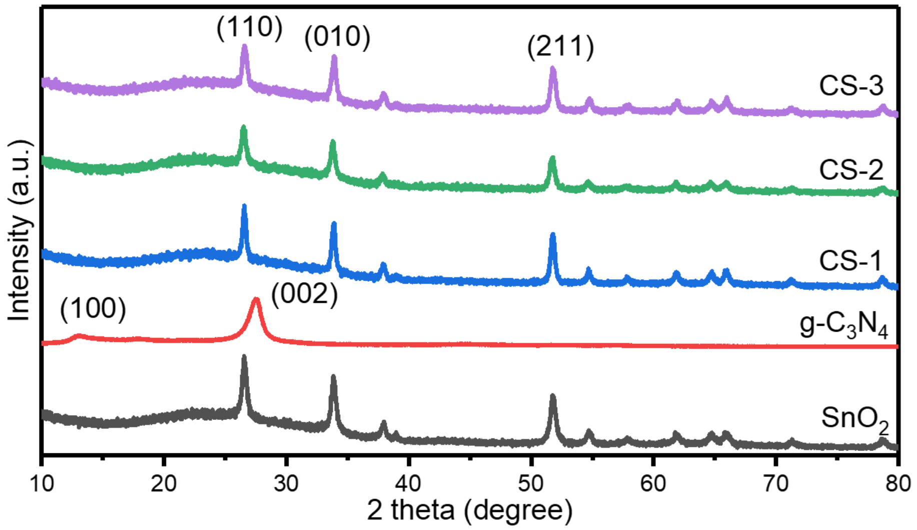

Figure S2. The XRD patterns of prepared samples.

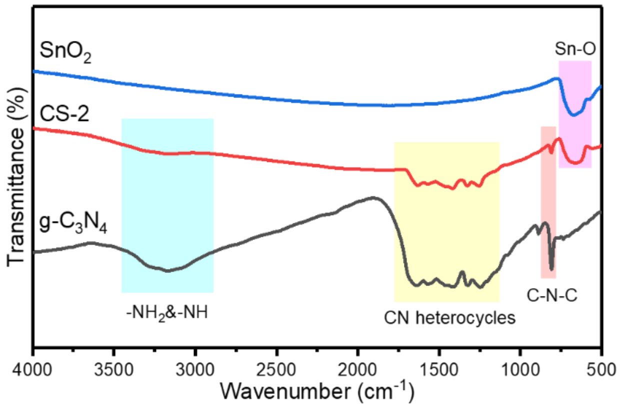

Figure S3. The FT-IR spectrum of g-C_3N_4, CS-2 and SnO_2.

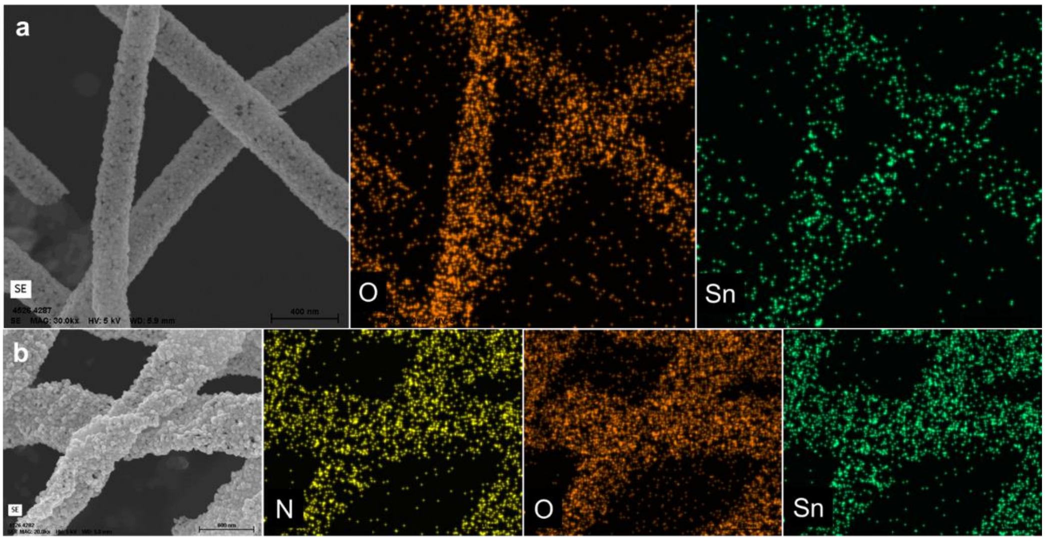

Figure S4. The elements mapping of (a) pristine SnO_2 and (b) CS-2.

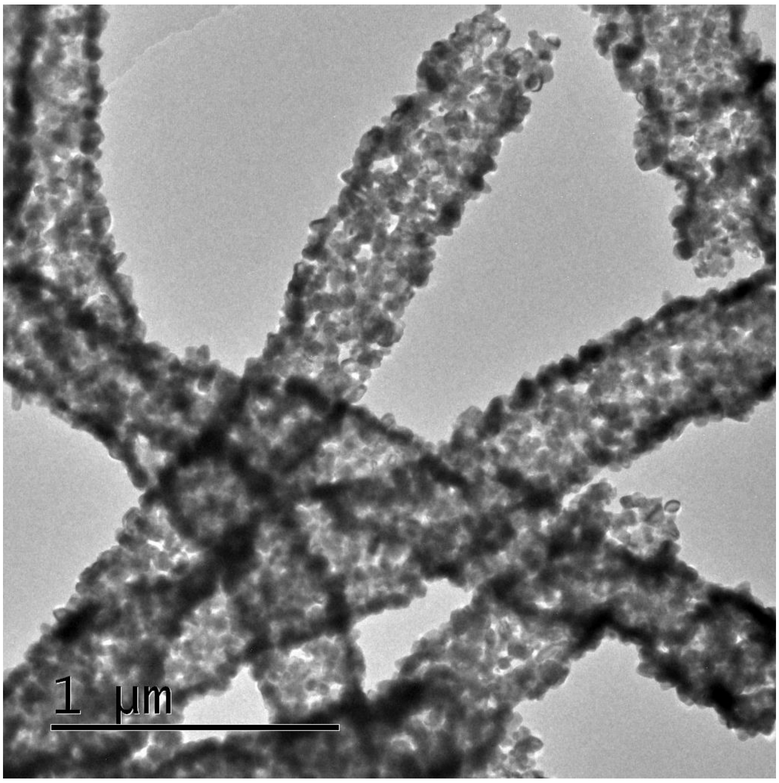

Figure S5. The TEM image of SnO_2.

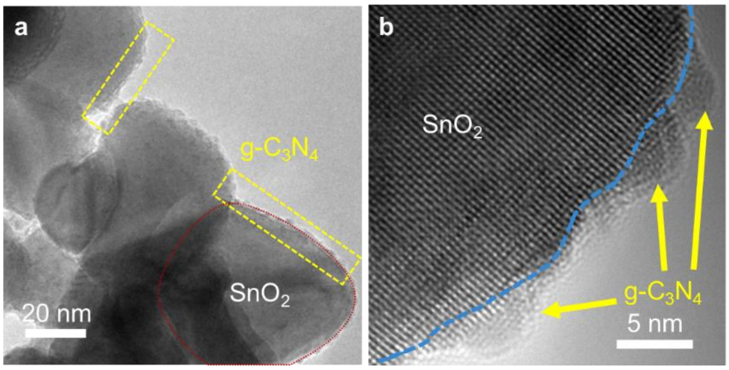

Figure S6. (a) and (b) HRTEM of CS-2.

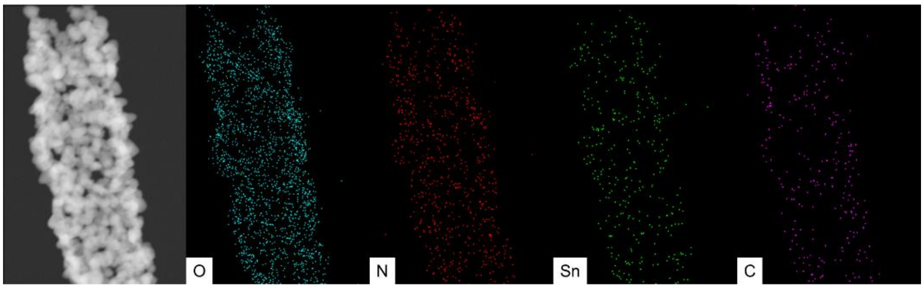

Figure S7. The element distribution of CS-2.

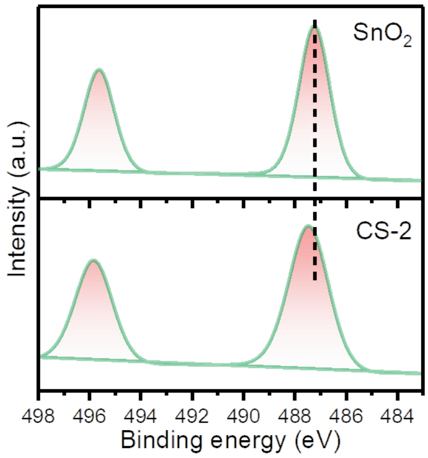

Figure S8. The comparison of Sn 3d in SnO_2 and CS-2.

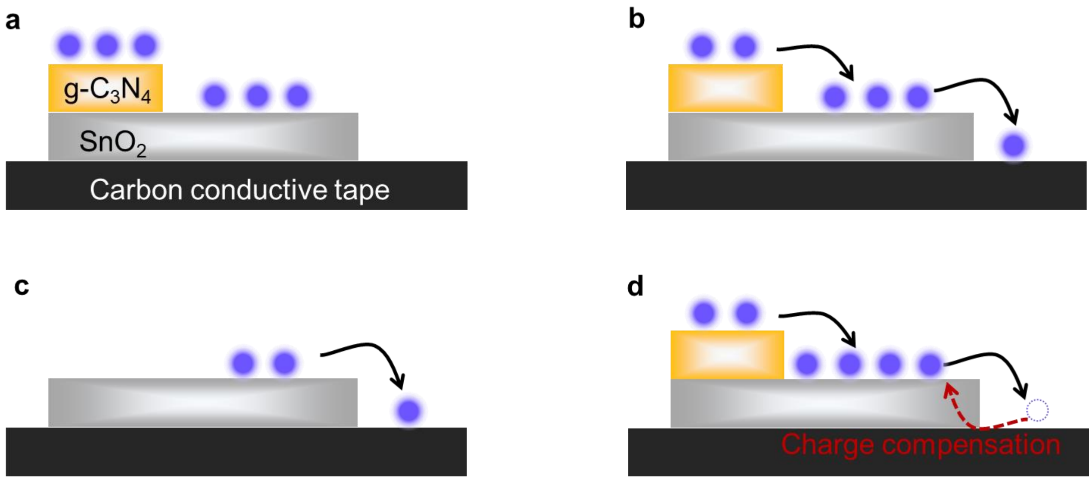

Figure S9. The charge transfers behaviors under different conditions: (a) no excitation and (b) under irradiation of CS-2, (c) under irradiation of SnO_2 and (d) charge compensation of CS-2.

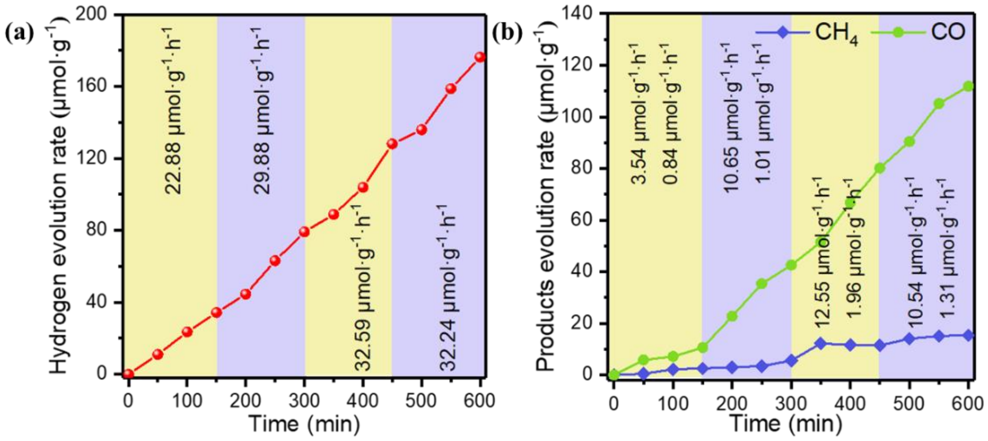

Figure S10. The cycling test of (a) water splitting and (b) CO_2 reduction photocatalytic reaction of CS-2.

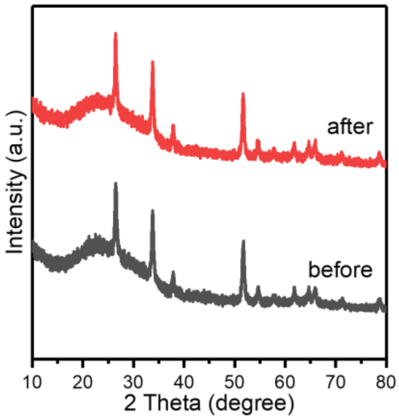

Figure S11. The XRD pattern of CS-2 before and after photocatalytic reaction.

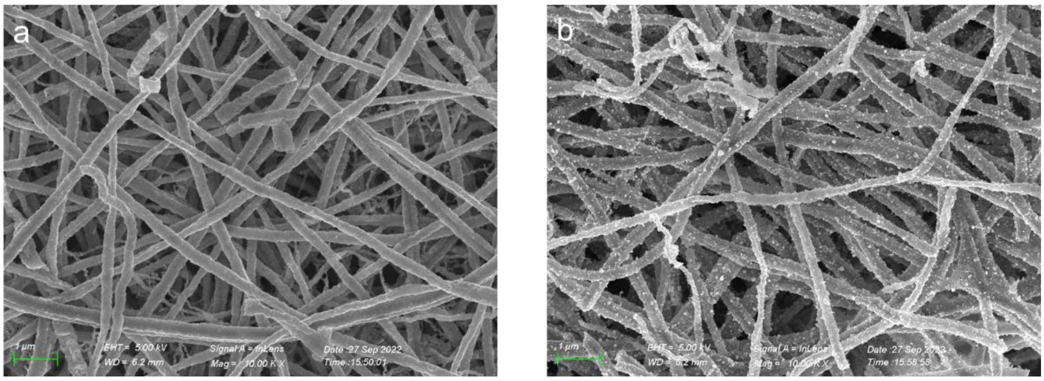

Figure S12. The SEM images of CS-2 before and after photocatalytic reaction.

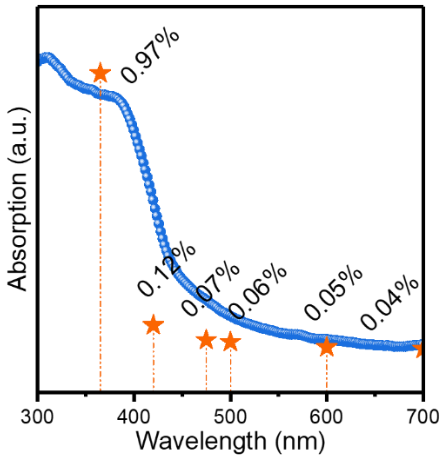

Figure S13. The AQE and UV-vis DRS spectrum of CS-2.

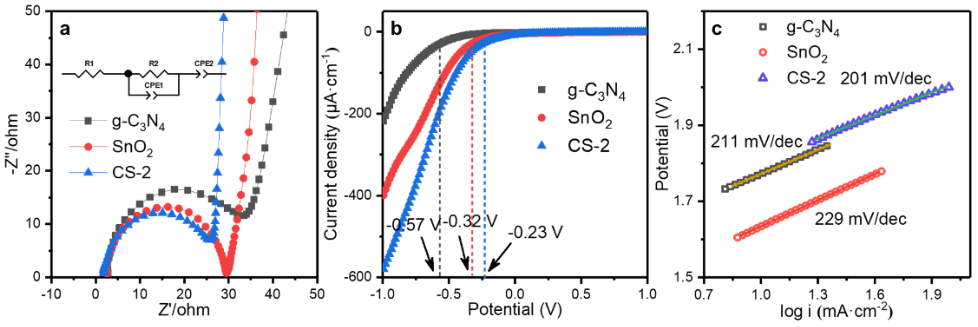

Figure S14. The (a) EIS curves (b) LSV curves and (c) Tafel slope of g-C_3N_4, SnO_2 and CS-2.

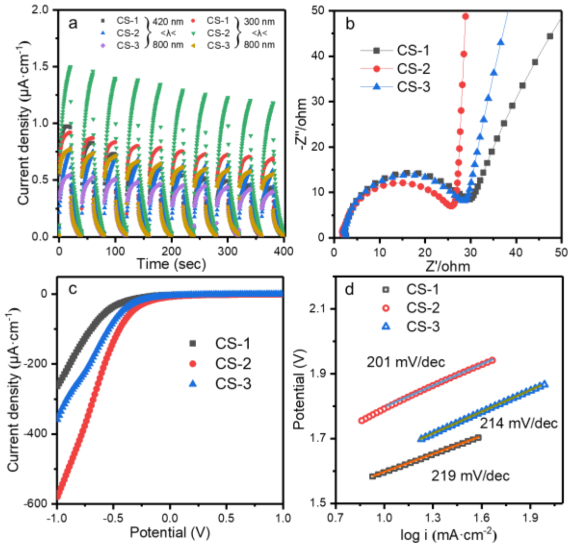

Figure S15. The (a) transient photocurrent response (b) EIS curves, (c) LSV curves and (d) Tafel slope of CS-1, CS-2 and CS-3.

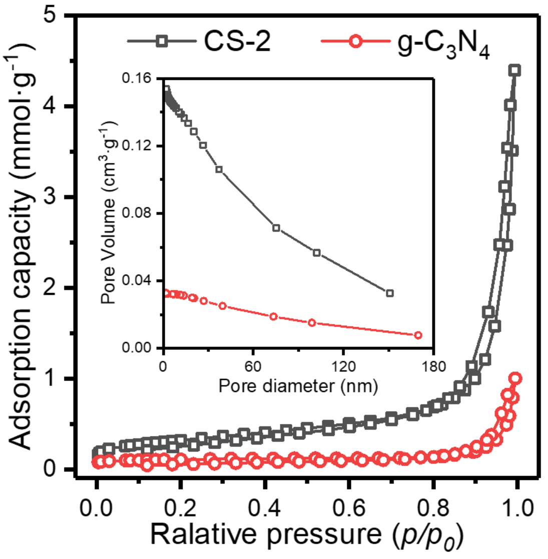

Figure S16. N_2 adsorption-desorption isotherms of CS-2 and g-C_3N_4.

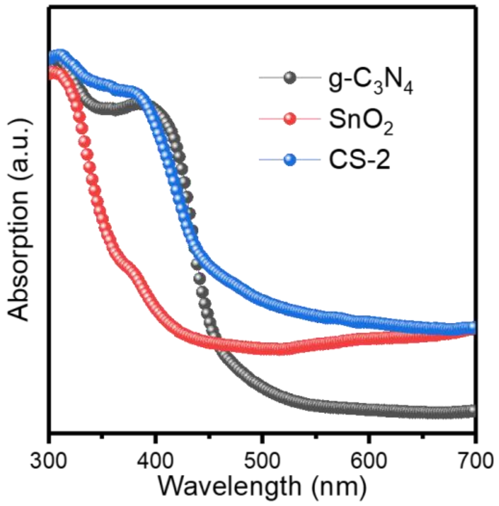

Figure S17. (a) The UV-vis DRS spectra and (b) the calculated band gap of g-C_3N_4, SnO_2 and CS-2.

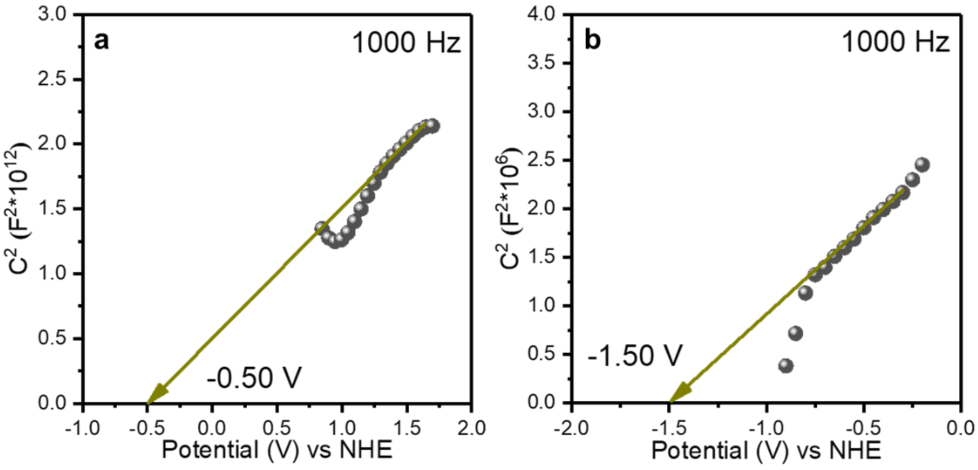

Figure S18. The Mott–Schottky curves of (a) SnO_2 and (b) g-C_3N_4.

Tables

Table S1. The mass ratio of different elements of SnO_2

Elements	Atomic number	quality [%]	Normalized quality [%]	Atomic ratio [%]	abs. error [%] (3 sigma)
C	6	45.27706	36.46971	71.41112	18.54178
N	7	0	0	0	0
O	8	15.62042	12.58192	18.49506	8.236928
Sn	50	63.25228	50.94837	10.09382	19.3226

Table S2. The mass ratio of different elements of CS-2

Elements	Atomic number	quality [%]	Normalized quality [%]	Atomic ratio [%]	abs. error [%] (3 sigma)
C	6	40.50557	34.52694	63.86104	16.20756
N	7	4.479257	3.818117	6.055787	3.378086
O	8	18.1093	15.43636	21.43376	8.737927
Sn	50	54.22173	46.21858	8.649409	16.26647

Table S3. The binding energy of Sn 3d in SnO_2 and CS-2 under different wavelength light irradiation

Wavelength [nm]	SnO_2		CS-2			
	Binding energy [eV]	Δ [eV]	Binding energy [eV]	Δ [eV]	Binding energy' [eV]	Δ' [eV]
Dark	487.16	0 (initial)	487.42	0 (initial)	487.42	0 (initial)
700 nm	487.19	-0.03	487.43	-0.01	487.40	0.02
650 nm	487.19	-0.03	487.43	-0.01	487.40	0.02
600 nm	487.16	0.00	487.39	0.03	487.39	0.03
550 nm	487.20	-0.04	487.42	0.00	487.38	0.04
500 nm	487.19	-0.03	487.40	0.02	487.37	0.05
450 nm	487.29	-0.13	487.41	0.01	487.28	0.14
400 nm	487.45	-0.29	487.43	-0.01	487.14	0.28
350 nm	487.51	-0.35	487.44	-0.02	487.09	0.33

Note: Binding energy' and Δ' were the results after deducting the substrate transfer.

Table S4. The parameters of different Sn compounds

Parameters	Sn-O	Sn-S	Sn-Cl	Sn-F	Sn-Br
a	487.27	491.60	487.30	488.59	487.56
b	2.44	6.77	2.47	3.76	2.73
c	0.70	0.92	0.53	0.62	0.49

Table S5. The electrons transfer numbers under different wavelength light irradiation of CS-2

Wavelength [nm]	Binding energy' [eV]	Δ' [eV]	Electron transfer numbers
Dark	487.42	0 (initial)	0
700 nm	487.40	0.02	0.05
650 nm	487.40	0.02	0.05
600 nm	487.39	0.03	0.07
550 nm	487.38	0.04	0.09
500 nm	487.37	0.05	0.11
450 nm	487.28	0.14	0.29
400 nm	487.14	0.28	0.53
350 nm	487.09	0.33	0.61

Table S6 The comparison of band position of several g-C_3N_4/SnO_2 composites.

Components	Position of CB [eV]		Position of VB [eV]		Ref
	g-C_3N_4	SnO_2	g-C_3N_4	SnO_2	
g-C_3N_4/SnO_2	-1.55	-0.44	1.19	2.94	This work
g-C_3N_4/$AgVO_3$/SnO_2	-1.19	-1.11	1.32	3.44	[S1]
g-C_3N_4/TiO_2/SnO_2	-1.12	-0.07	1.57	3.53	[S2]
g-C_3N_4/ZnO/SnO_2	-1.2	-0.1	1.5	3.4	[S3]
g-C_3N_4/SnO_2	-0.9	-0.07	1.88	3.68	[S4]
g-C_3N_4/SnO_2	-1.14	0.05	1.58	3.45	[S5]
g-C_3N_4/CDs/SnO_2	-0.93	0.05	1.75	3.48	[S6]
g-C_3N_4/SnO_2	-1.13	0.05	1.57	3.48	[S7]

[S1] G. Koyyada, N. Siva Kumar, E. H. Al-Ghurabi, M. Asif, K. Mallikarjuna, *Environmental Science and Pollution Research* 2021, 28, 31585.

[S2] M. Huang, J. Li, Y. Huang, X. Zhou, Z. Qin, Z. Tong, M. Fan, B. Li, L. Dong, *Journal of Alloys and Compounds* 2021, 864, 158132.

[S3] S. V. P. Vattikuti, P. A. K. Reddy, J. Shim, C. Byon, *ACS Omega* 2018, 3, 7587.

[S4] M. Ismael, E. Elhaddad, M. Wark, *Colloids and Surfaces A: Physicochemical and Engineering Aspects* 2022, 638, 128288.

[S5] K. N. Van, H. T. Huu, V. N. Nguyen Thi, T. L. Le Thi, D. H. Truong, T. T. Truong, N. N. Dao, V. Vo, D. L. Tran, Y. Vasseghian, *Chemosphere* 2022, 289, 133120.

[S6] D. Li, J. Huang, R. Li, P. Chen, D. Chen, M. Cai, H. Liu, Y. Feng, W. Lv, G. Liu, *Journal of Hazardous Materials* 2021, 401, 123257.

[S7] X. Wang, P. Ren, *Advanced Powder Technology* 2018, 29, 1153.
