# Supplementary material for: Detecting and Quantifying Wavelength‐Dependent Electrons Transfer in Heterostructure Catalyst via In Situ Irradiation XPS
Source: Adv Sci (Weinh). 2022 Nov 14;10(4):2205020. doi: 10.1002/advs.202205020 (PMC9896054; doi:10.1002/advs.202205020)
Supplement: Supplementary file 2 — Supporting Information [file ADVS-10-2205020-s001.pptx]

## Slide 1
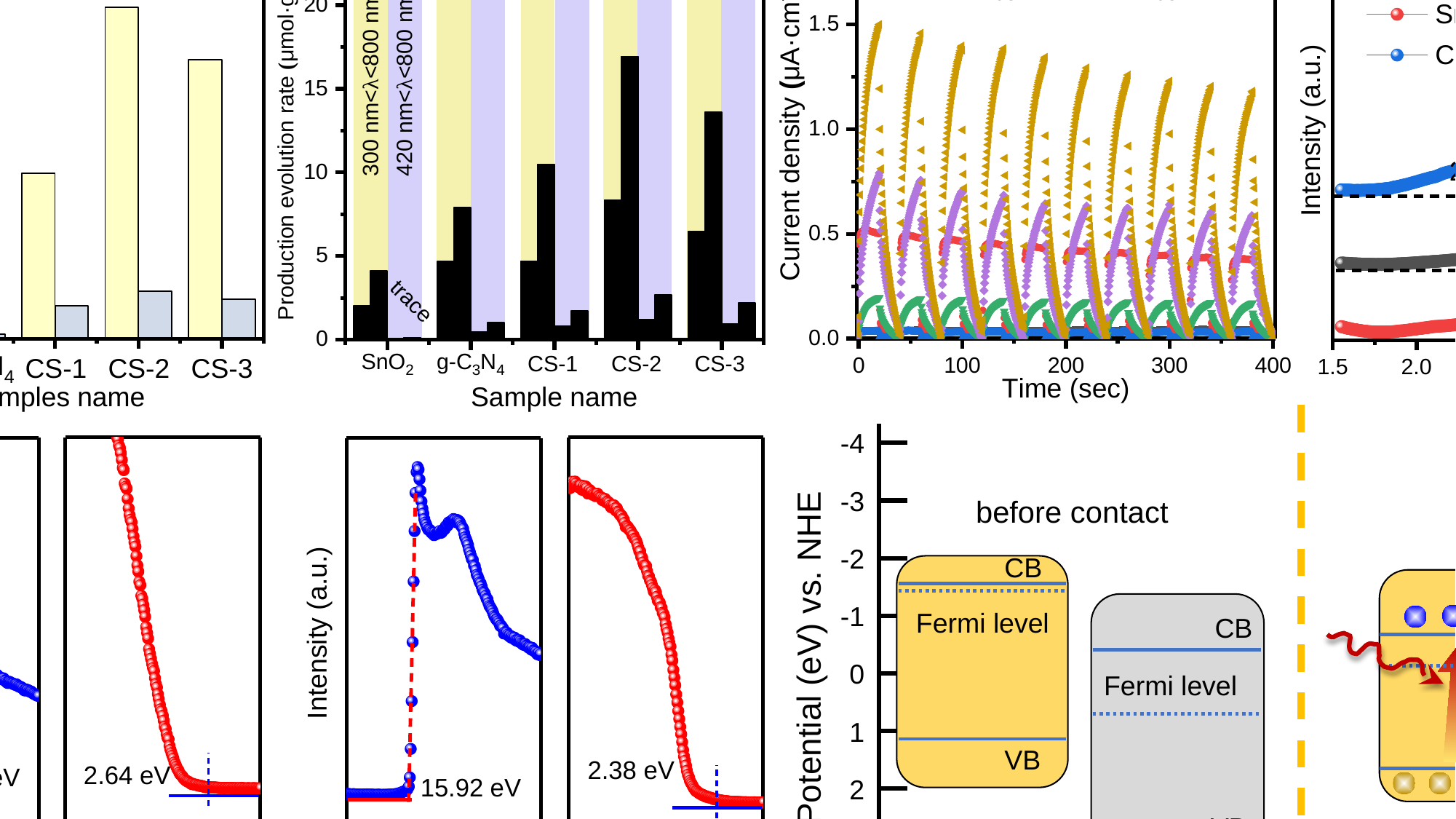

-4
-3
before contact
after contact
-2
CB
-1
Fermi level
CB
Intensity (a.u.)
Intensity (a.u.)
CB
Potential (eV) vs. NHE
0
Fermi level
Fermi level
1
VB
2
VB
g-C3N4
VB
3
Binding energy (eV)
Binding energy (eV)
4
SnO2

## Slide 2
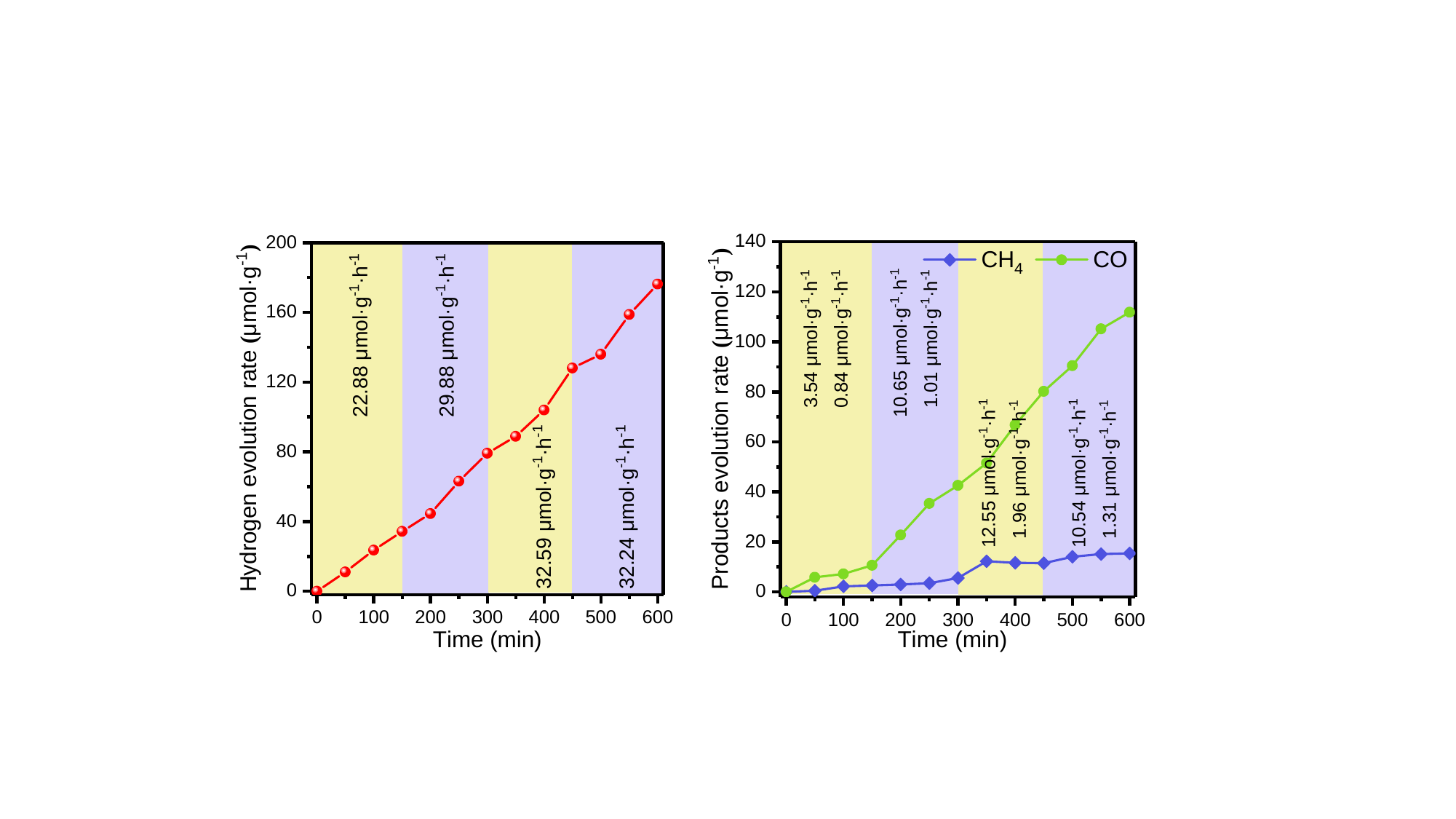

## Slide 3
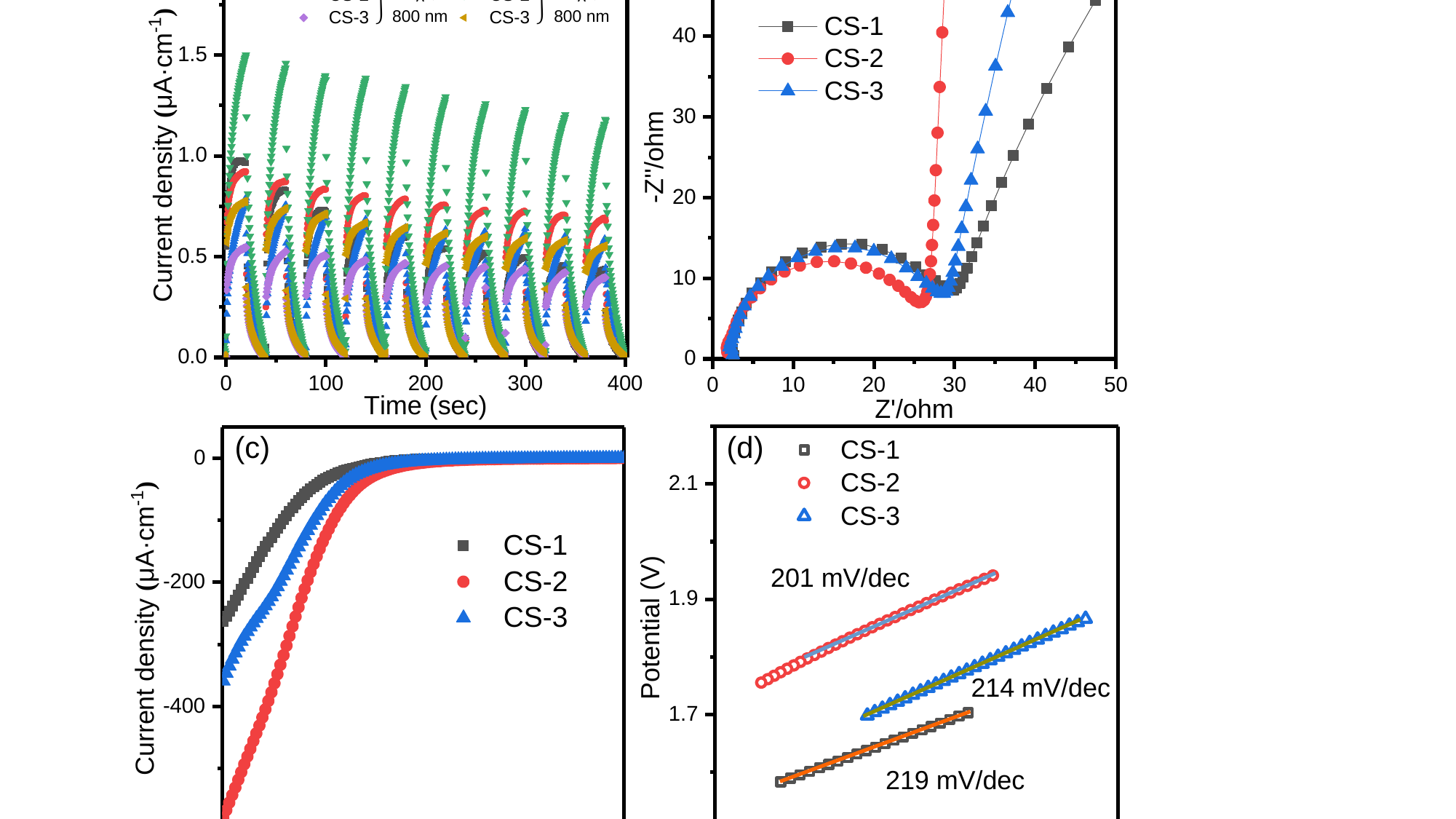

(b)
(a)
(d)
(c)

## Slide 4
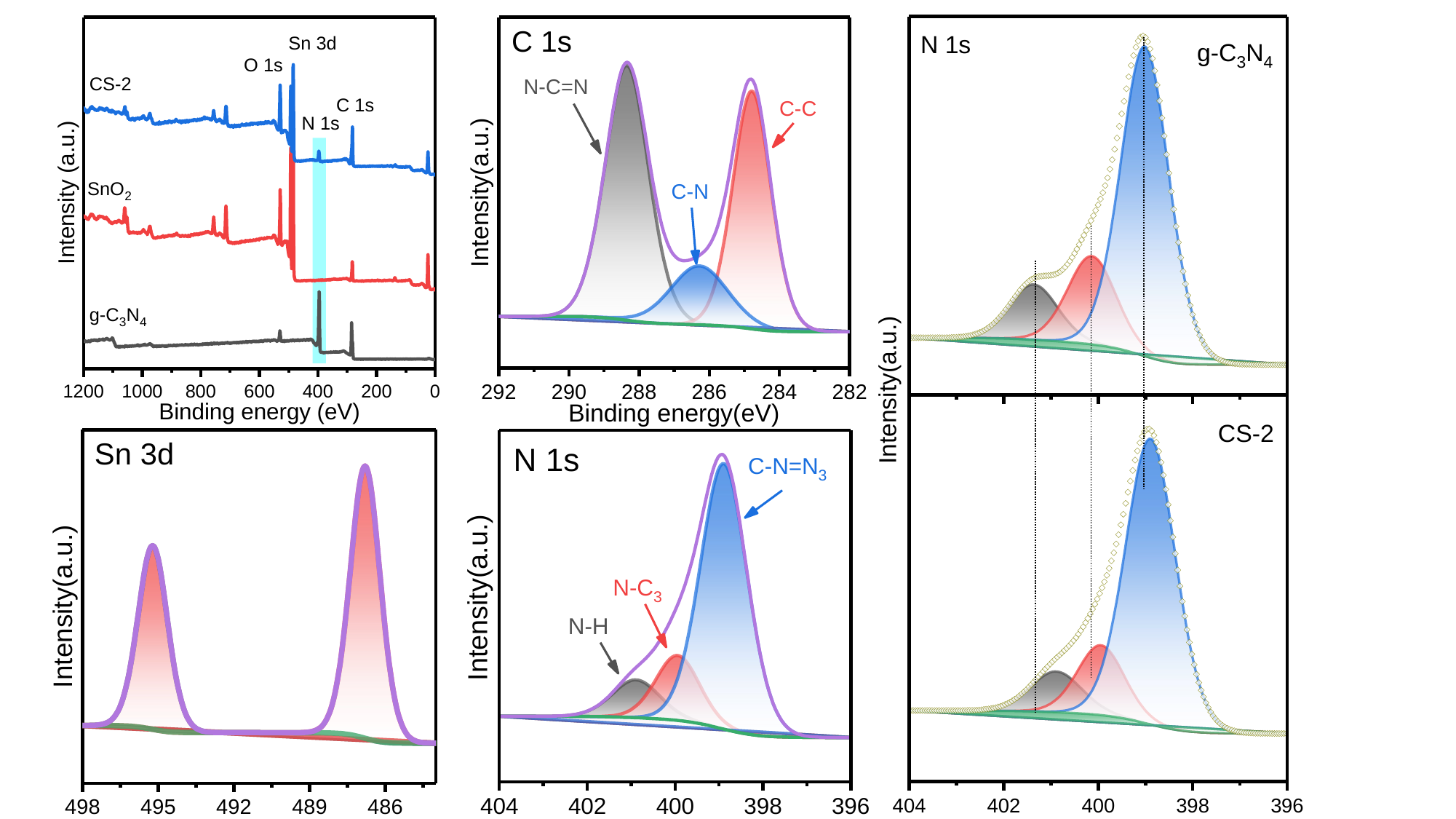

## Slide 5
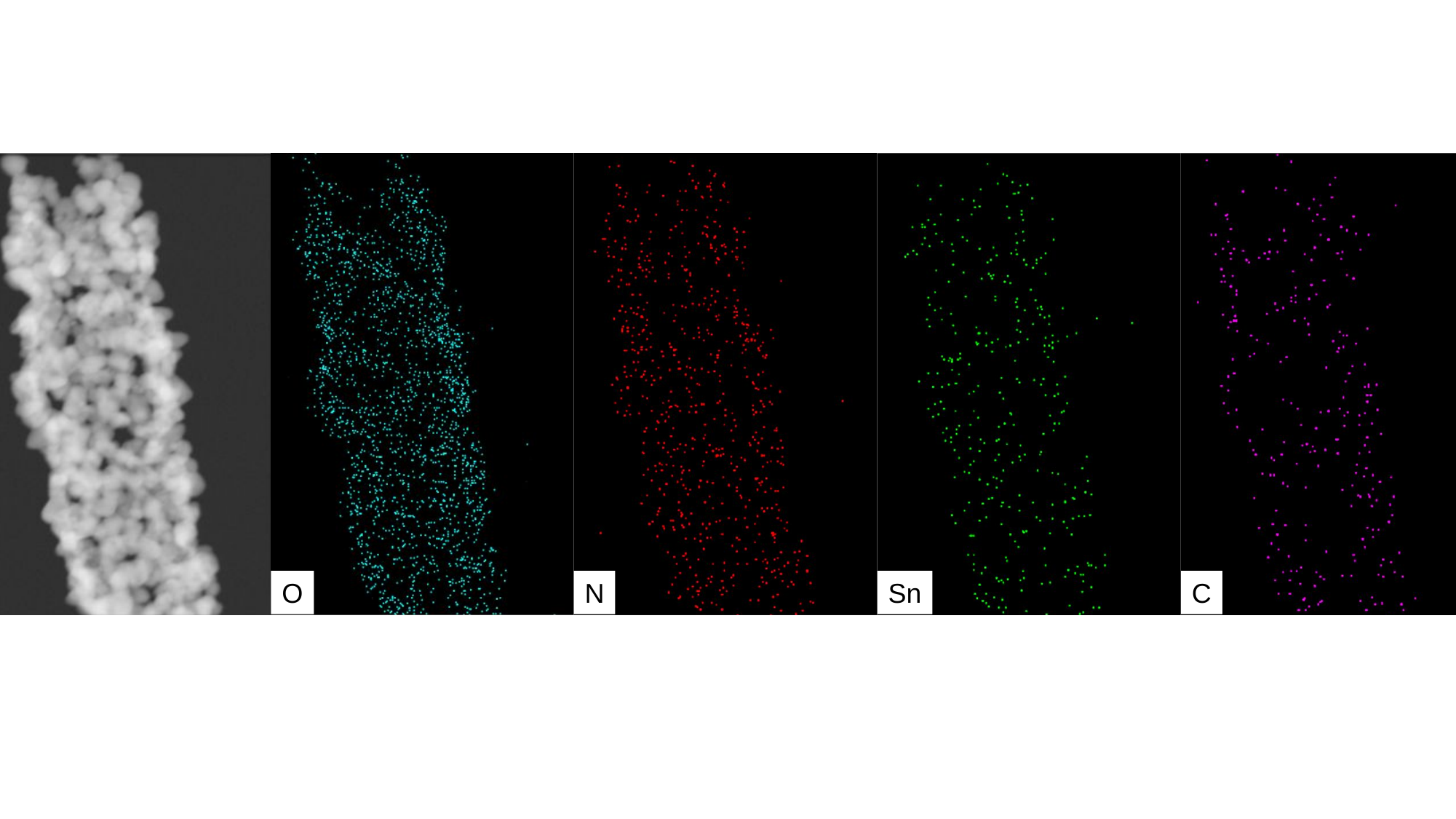

O
N
Sn
C

## Slide 6
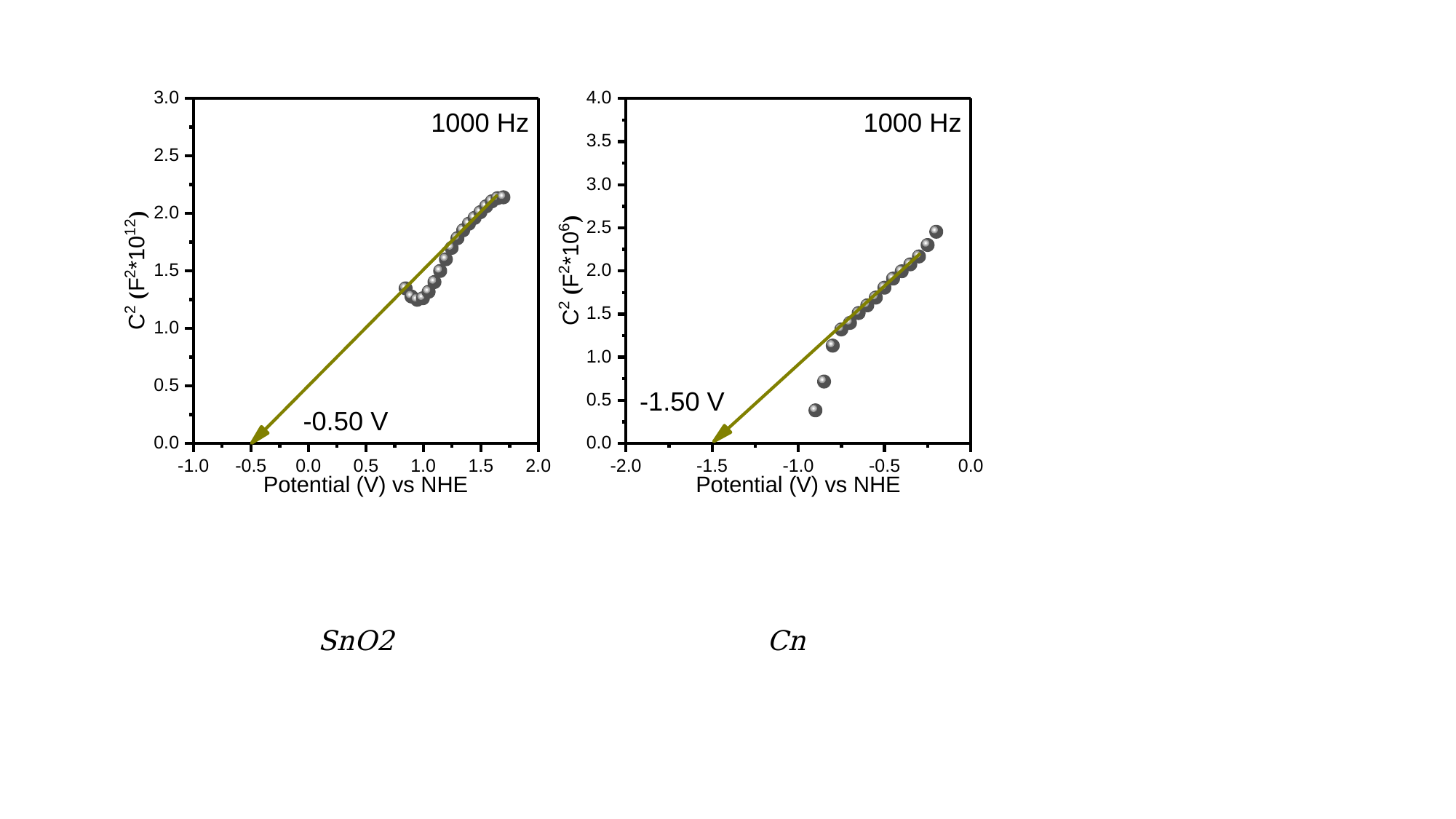

SnO2
Cn

## Slide 7
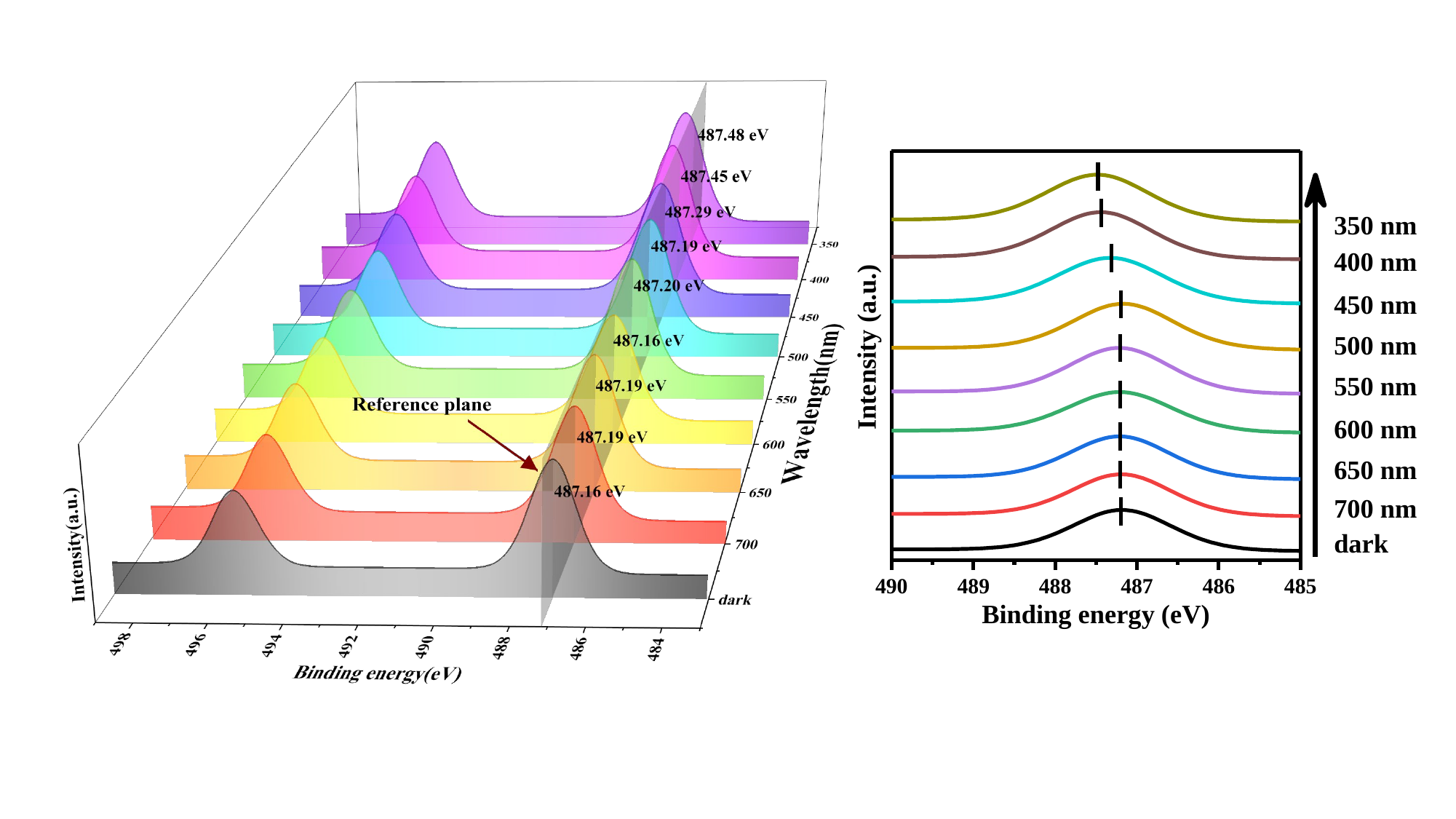

## Slide 8
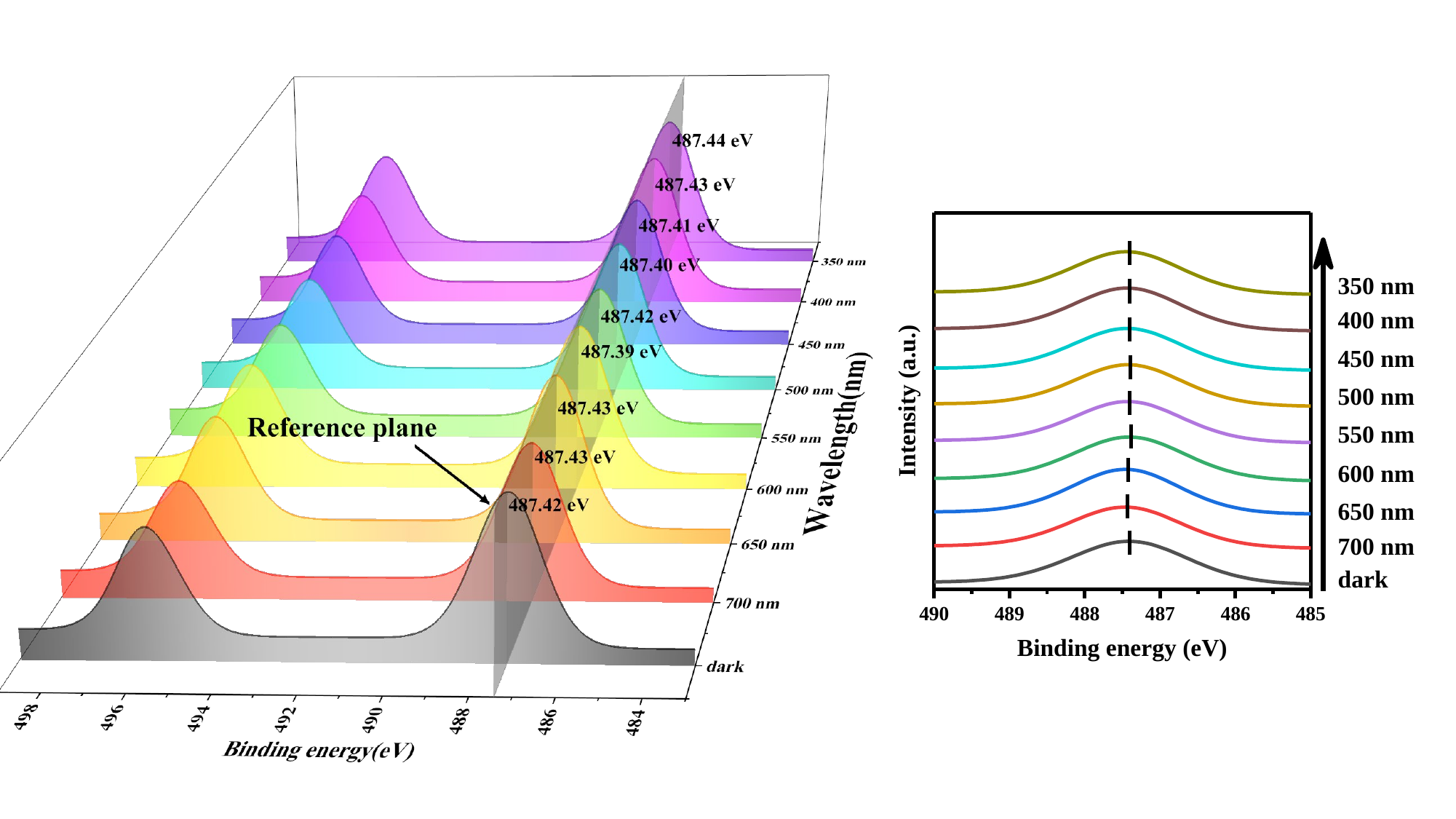

## Slide 9
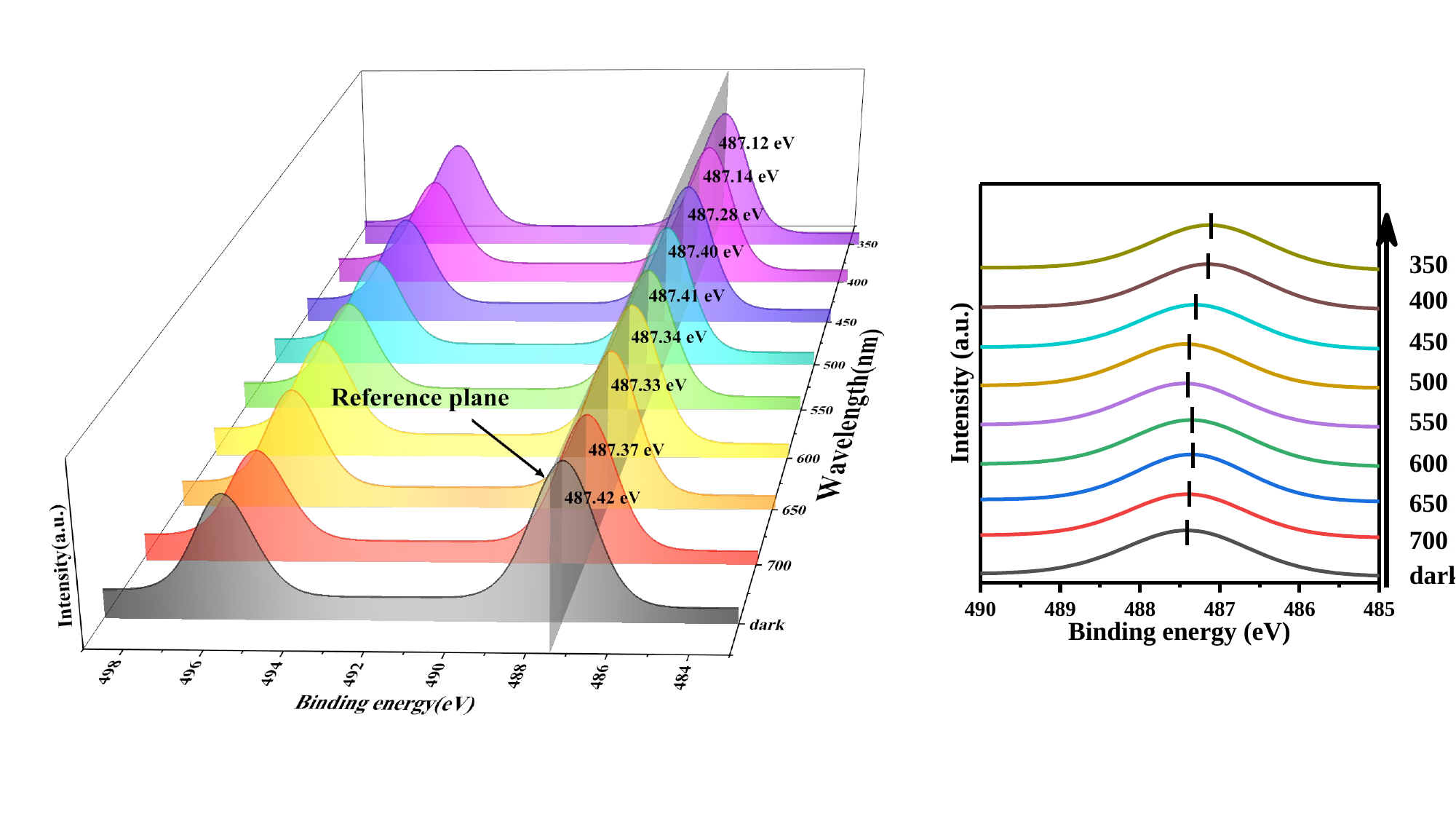

## Slide 10
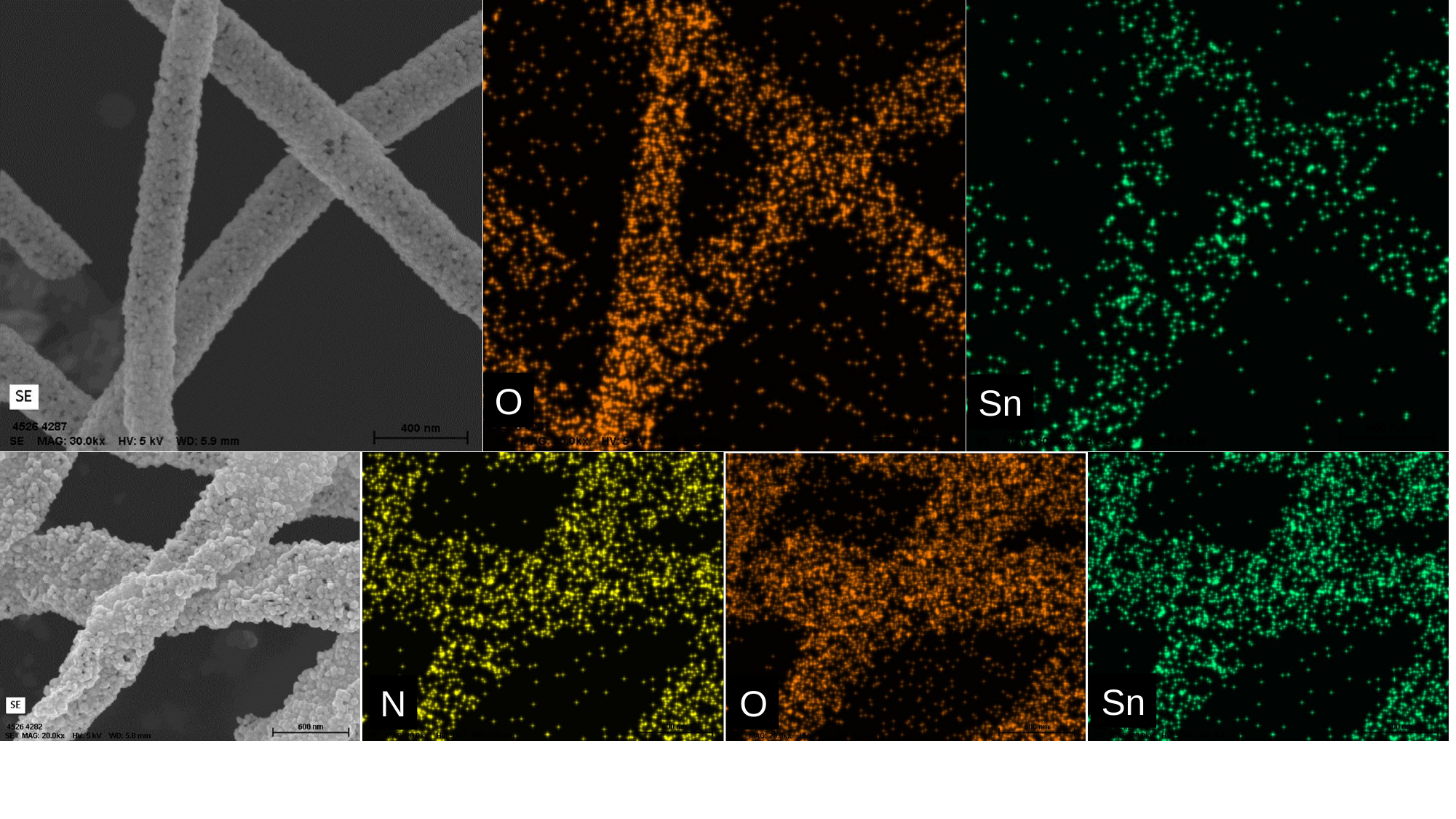

O
Sn
Sn
N
O

## Slide 11
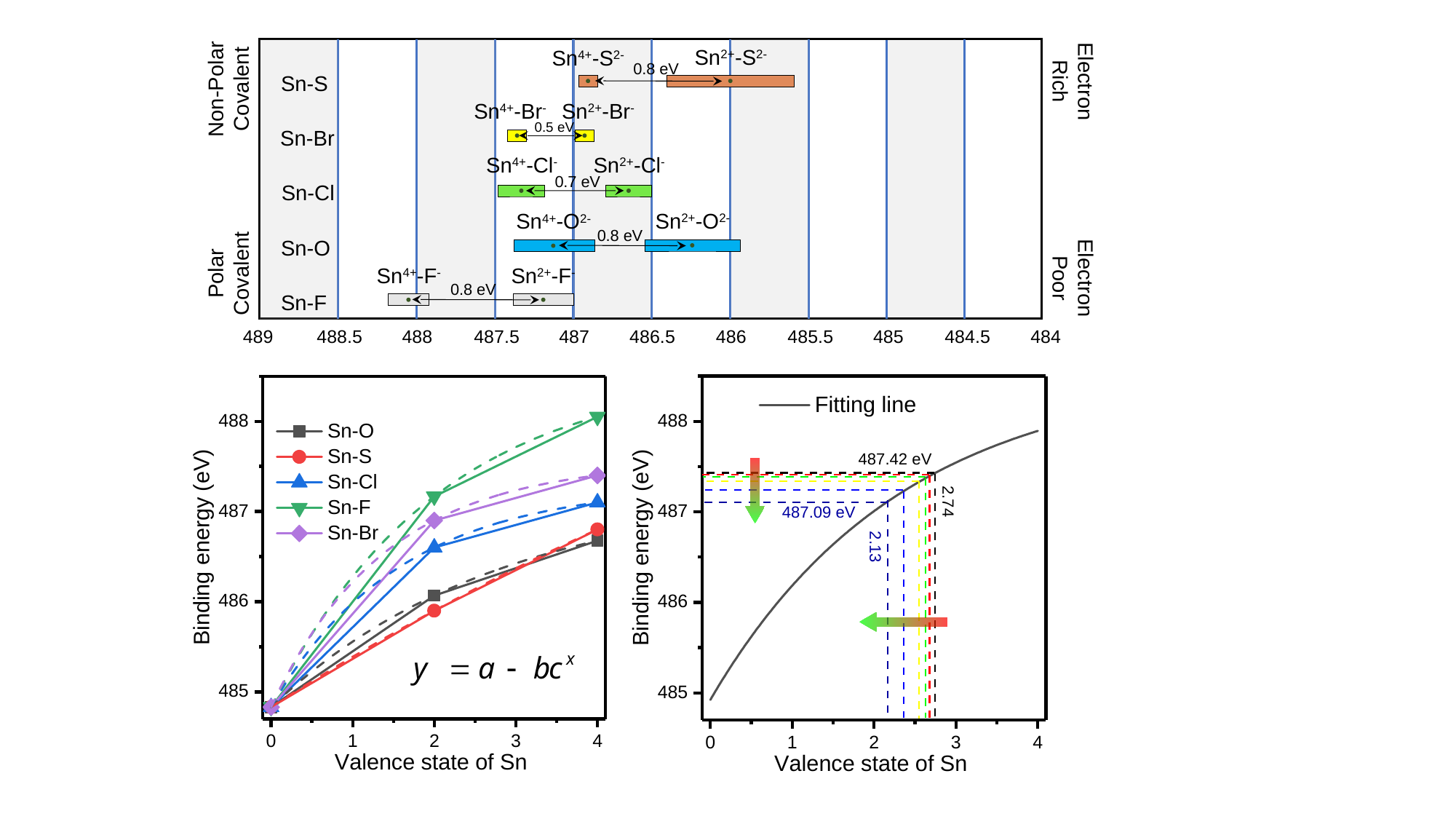

Sn2+-S2-
Sn4+-S2-
Electron
Rich
0.8 eV
Non-Polar
Covalent
Sn-S
Sn4+-Br-
Sn2+-Br-
0.5 eV
Sn-Br
Sn4+-Cl-
Sn2+-Cl-
0.7 eV
Sn-Cl
Sn2+-O2-
Sn4+-O2-
0.8 eV
Sn-O
Polar
Covalent
Electron
Poor
Sn4+-F-
Sn2+-F-
0.8 eV
Sn-F
489
488.5
488
487.5
487
486.5
486
485.5
485
484.5
484

## Slide 12
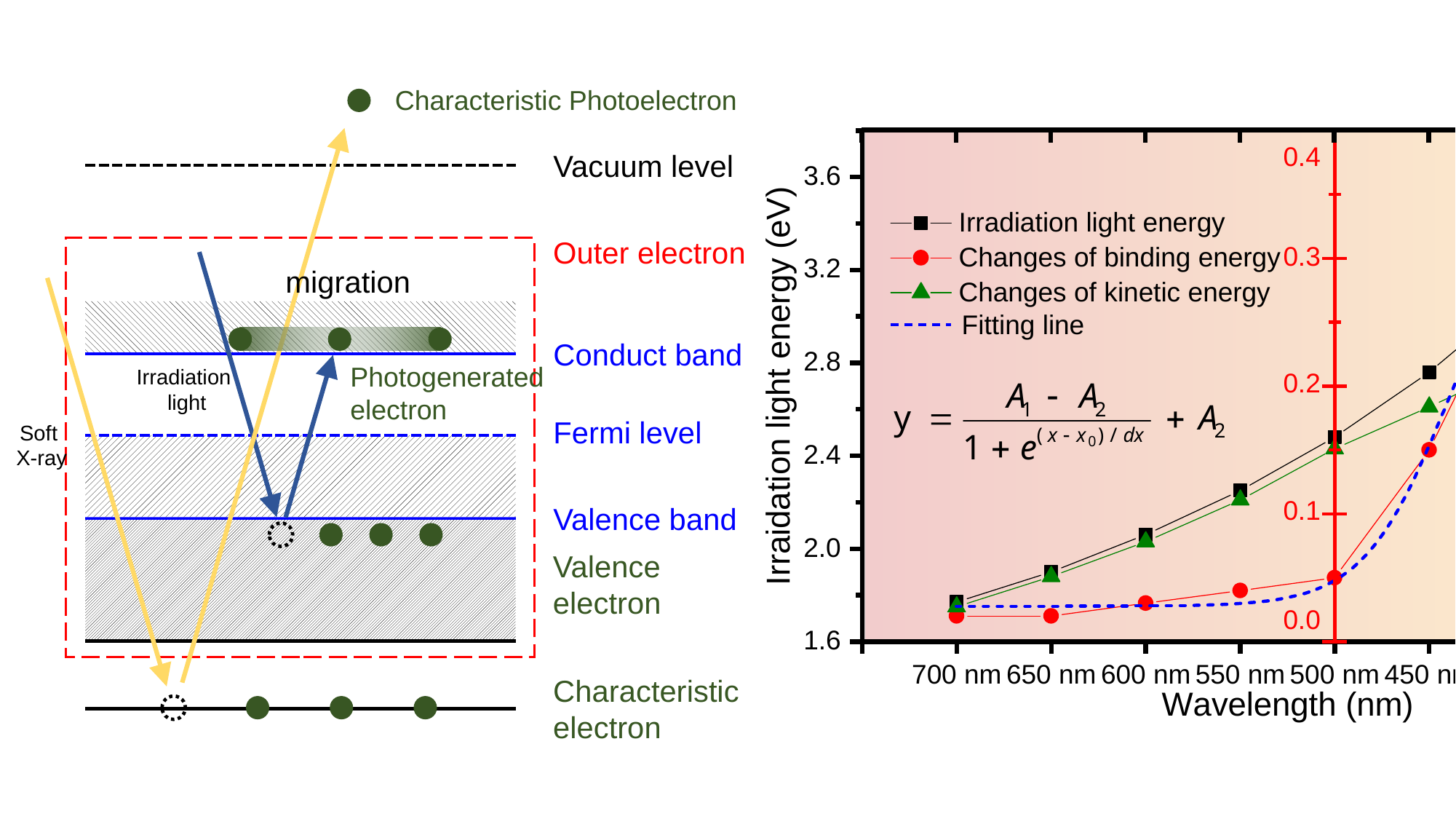

Characteristic Photoelectron
Vacuum level
Outer electron
migration
Conduct band
Photogenerated
electron
Irradiation
 light
Fermi level
Soft
 X-ray
Valence band
Valence
electron
Characteristic
electron

## Slide 13
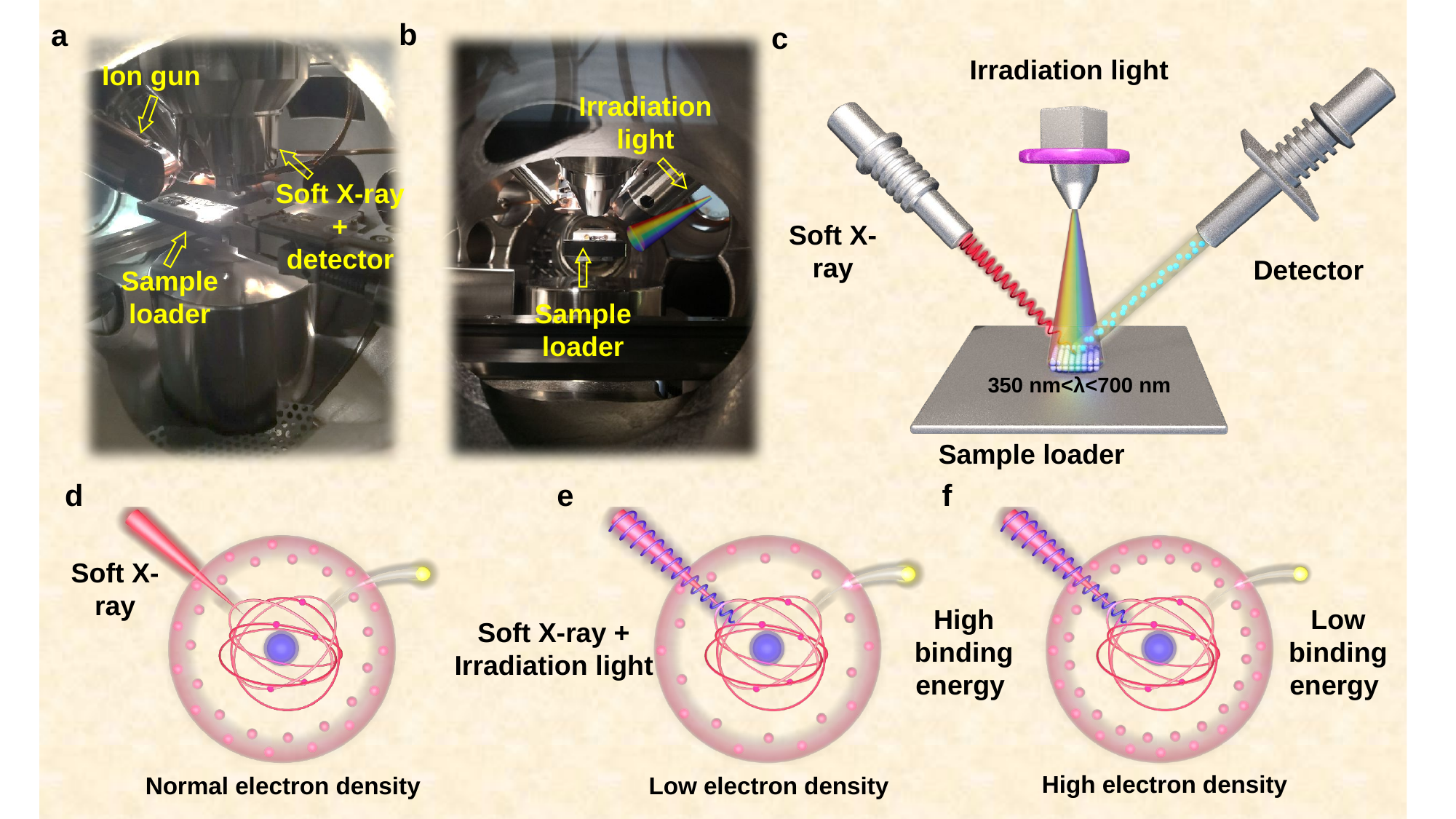

b
a
c
Irradiation light
Ion gun
Irradiation light
Soft X-ray +
detector
Soft X-ray
Detector
Sample loader
Sample loader
350 nm<λ<700 nm
Sample loader
d
e
f
Soft X-ray
High binding energy
Low binding energy
Soft X-ray +
Irradiation light
High electron density
Normal electron density
Low electron density

## Slide 14
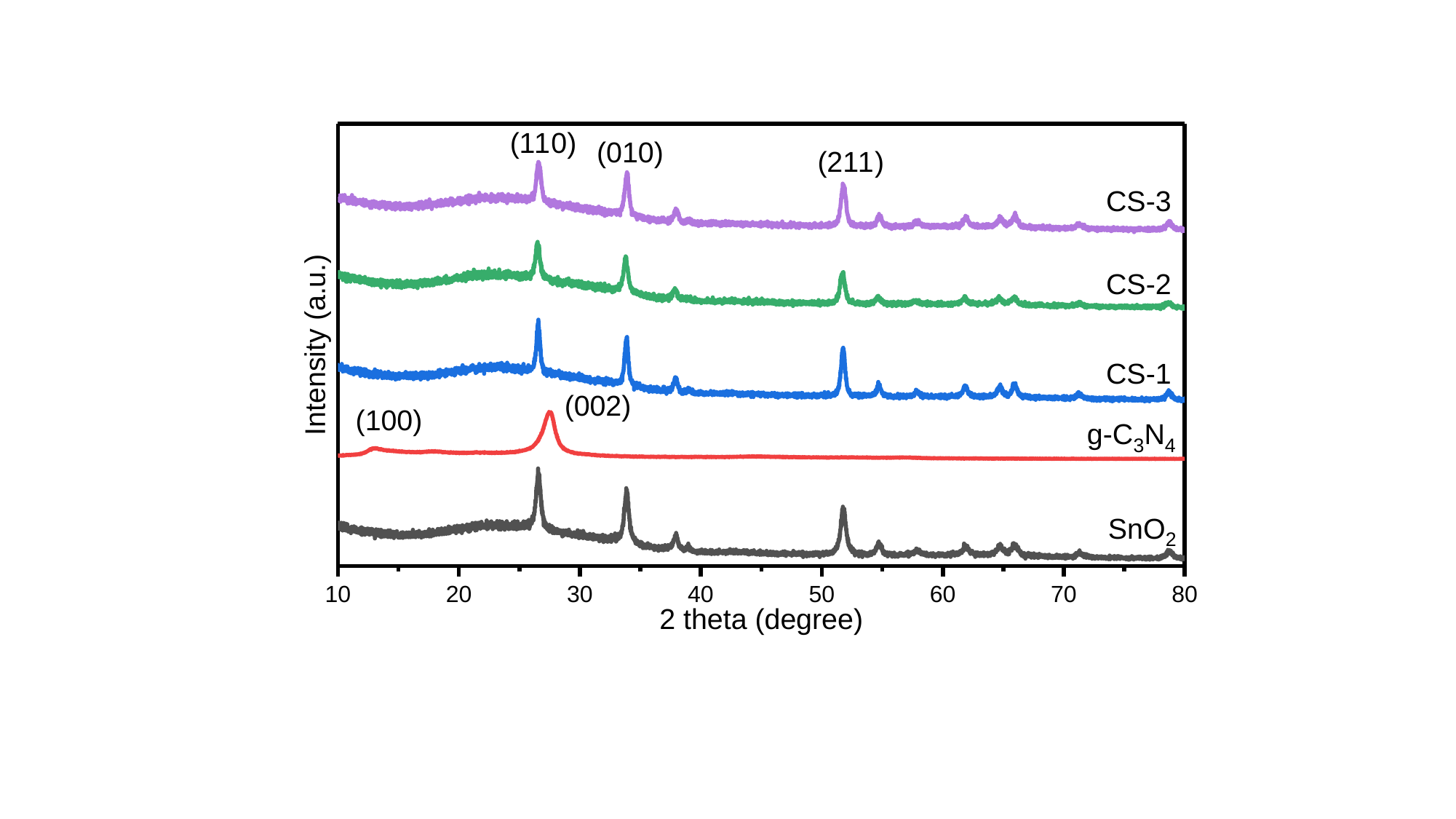

## Slide 15
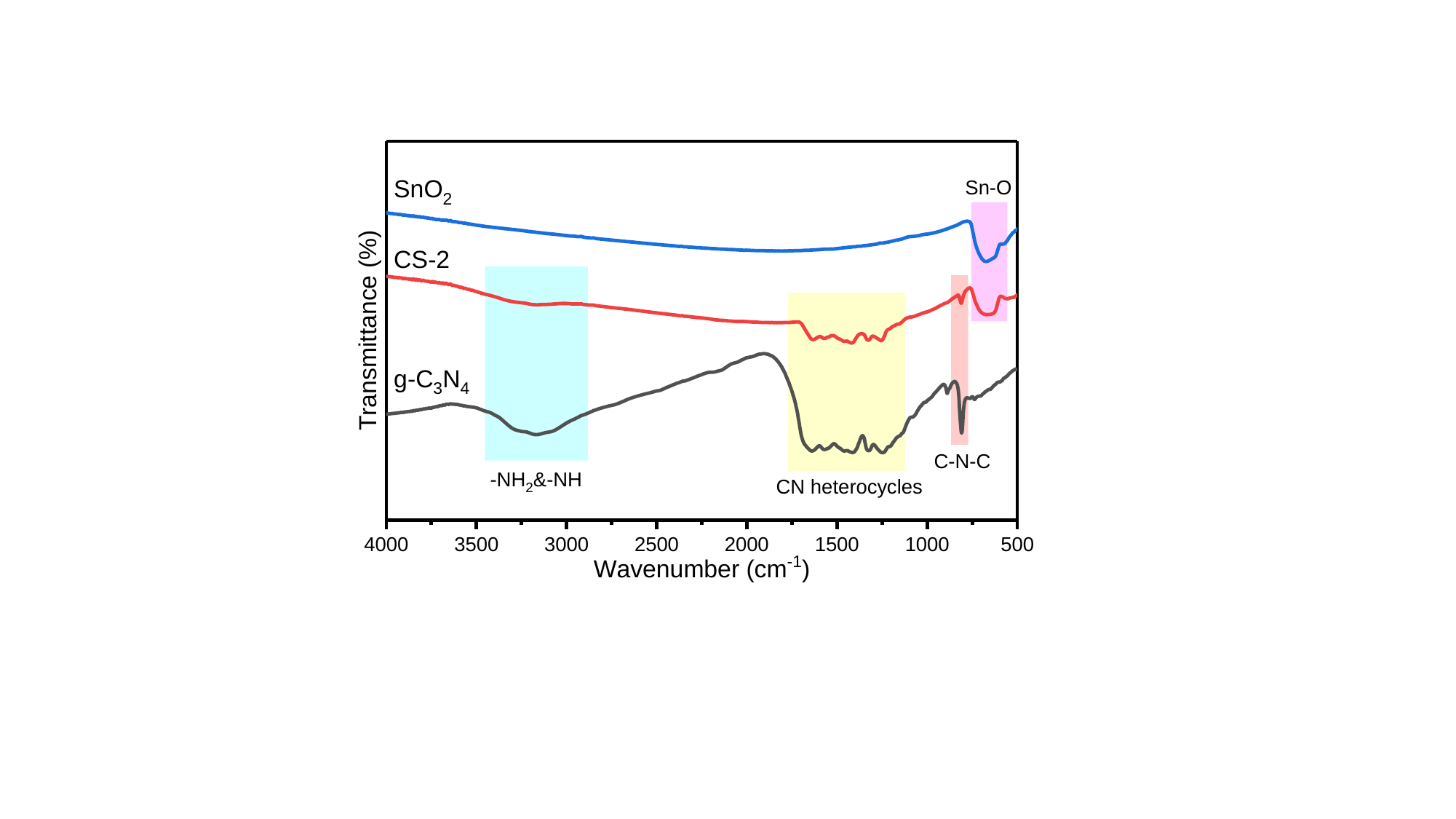

## Slide 16
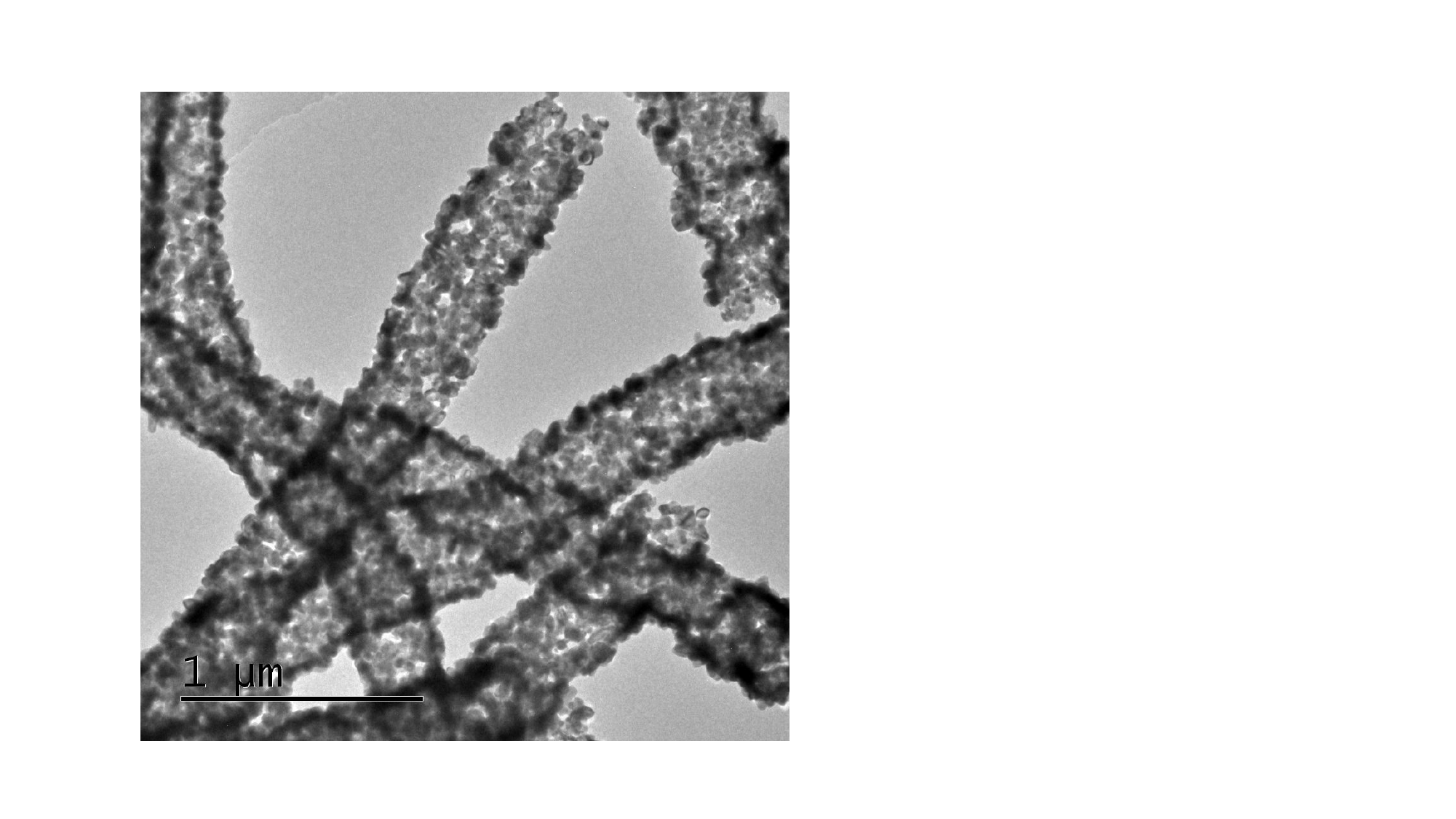

## Slide 17
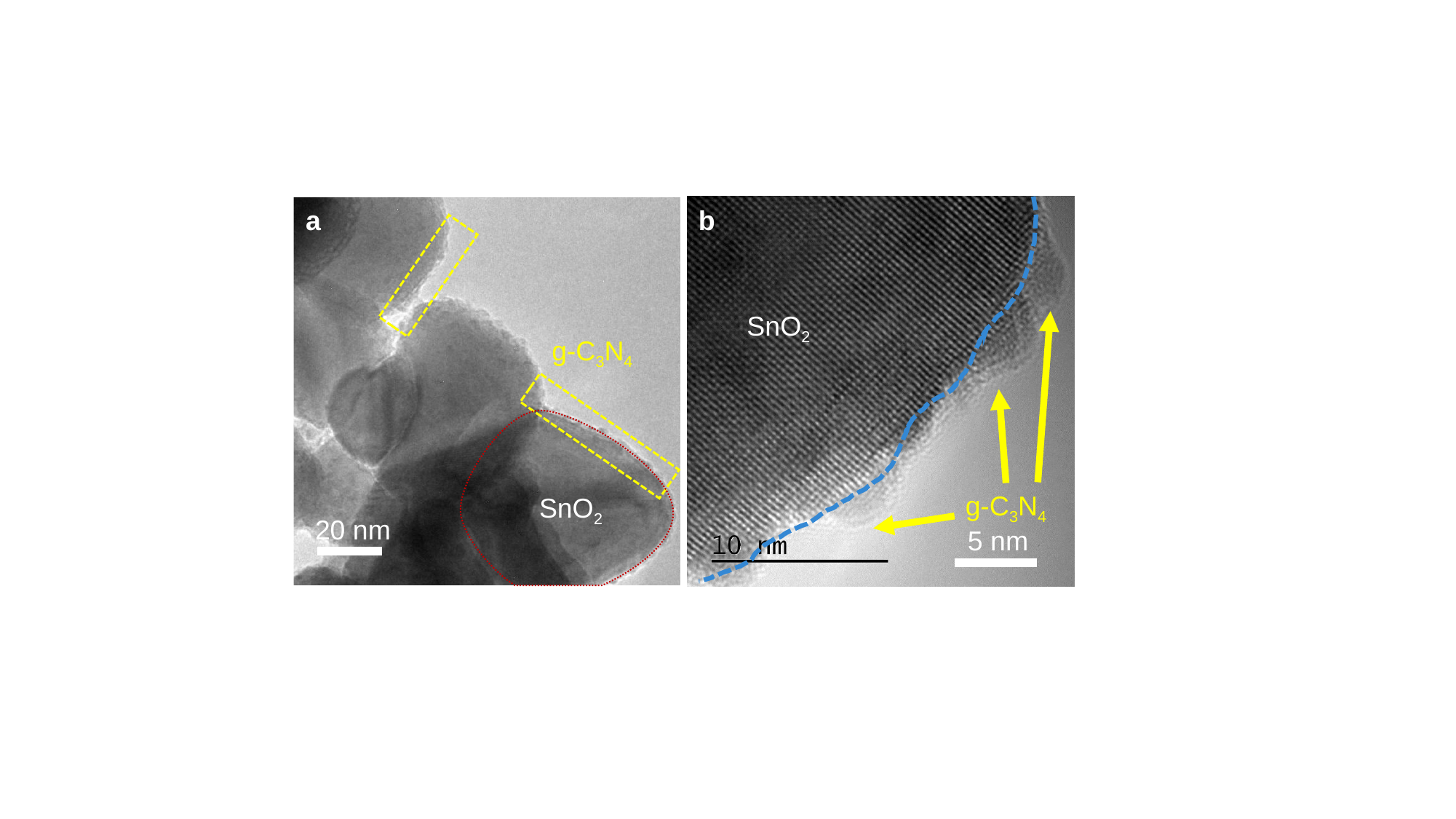

SnO2
g-C3N4
5 nm
a
b
g-C3N4
SnO2
20 nm

## Slide 18
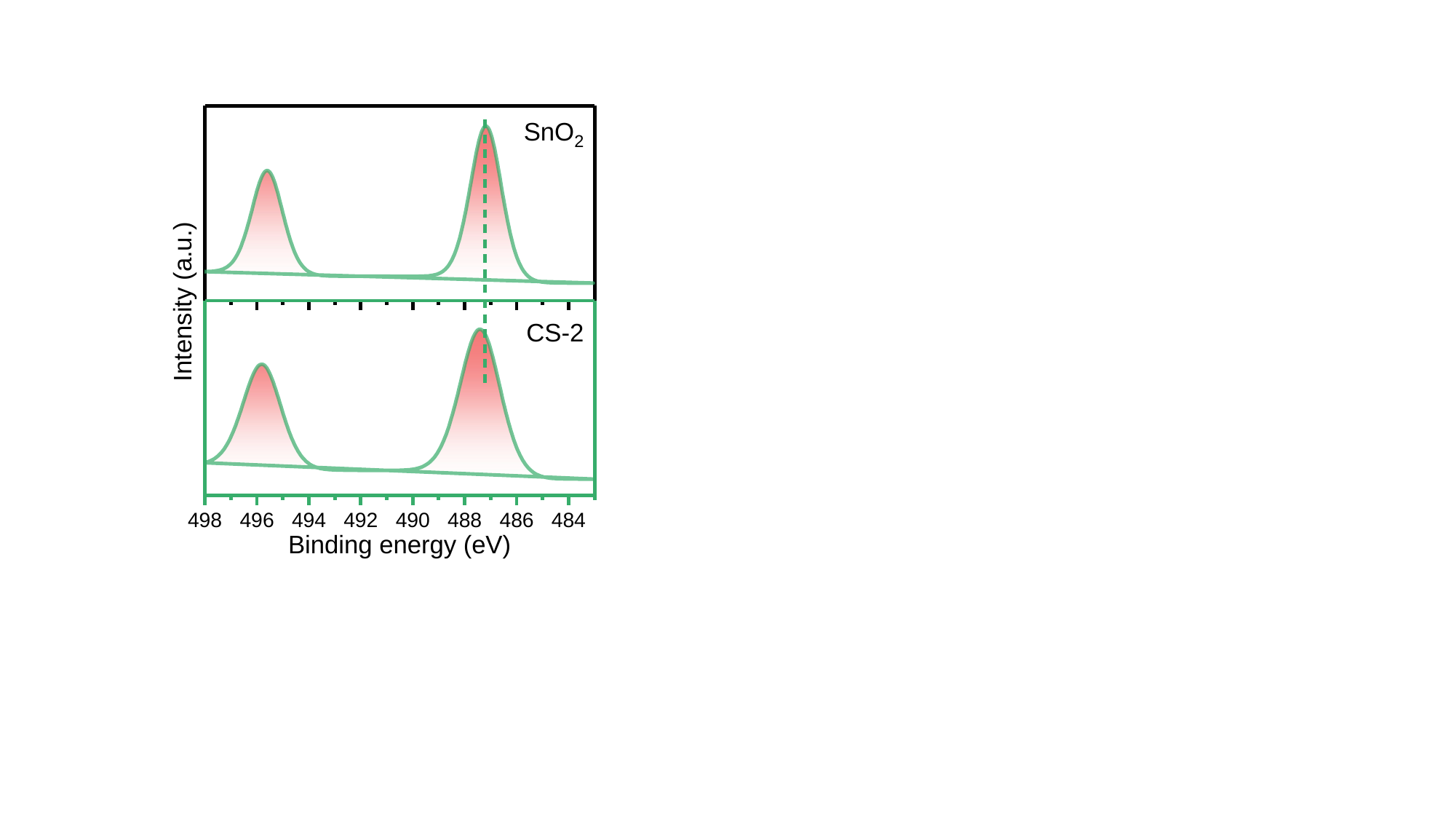

## Slide 19
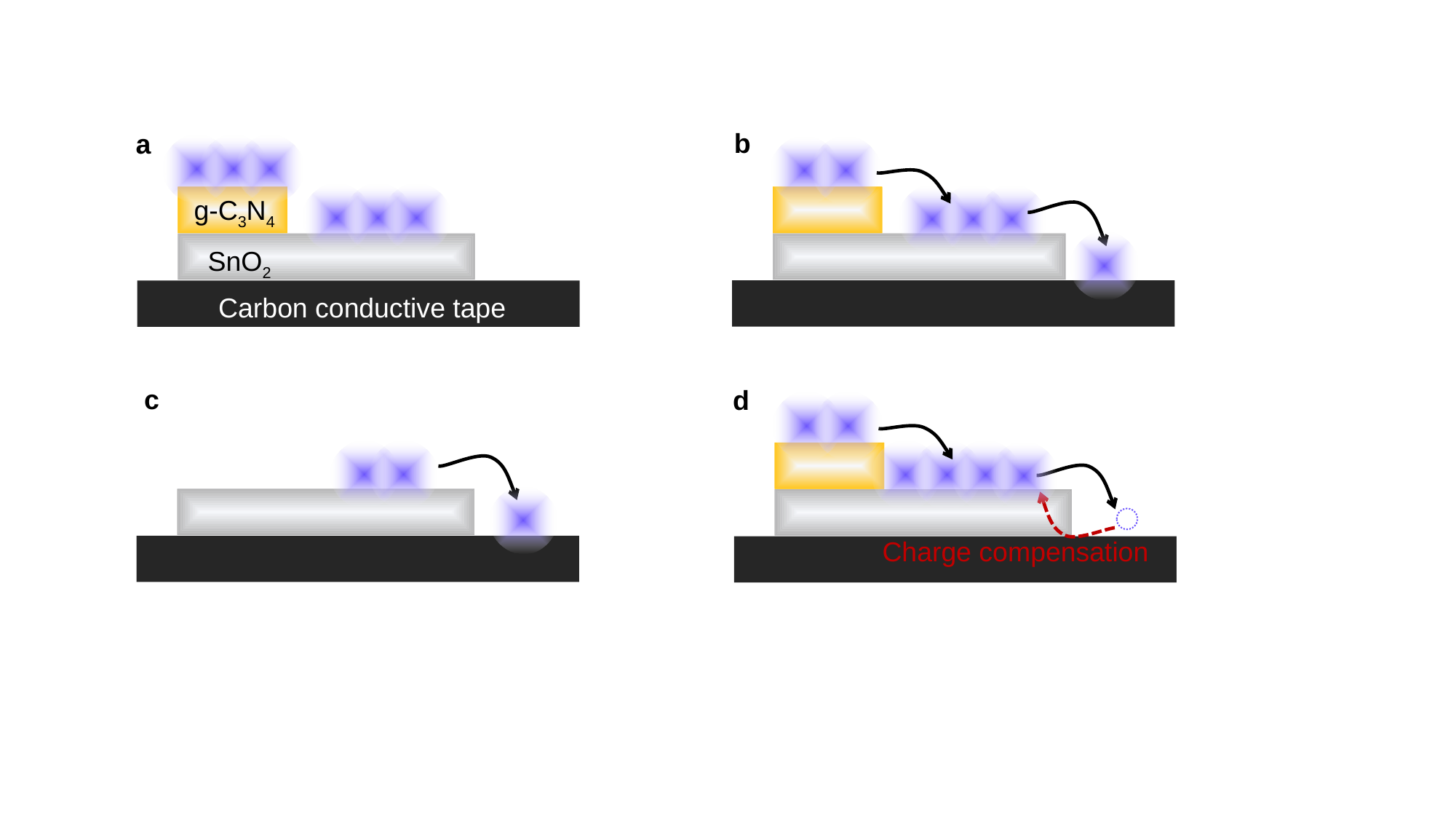

b
a
g-C3N4
SnO2
Carbon conductive tape
c
d
Charge compensation

## Slide 20
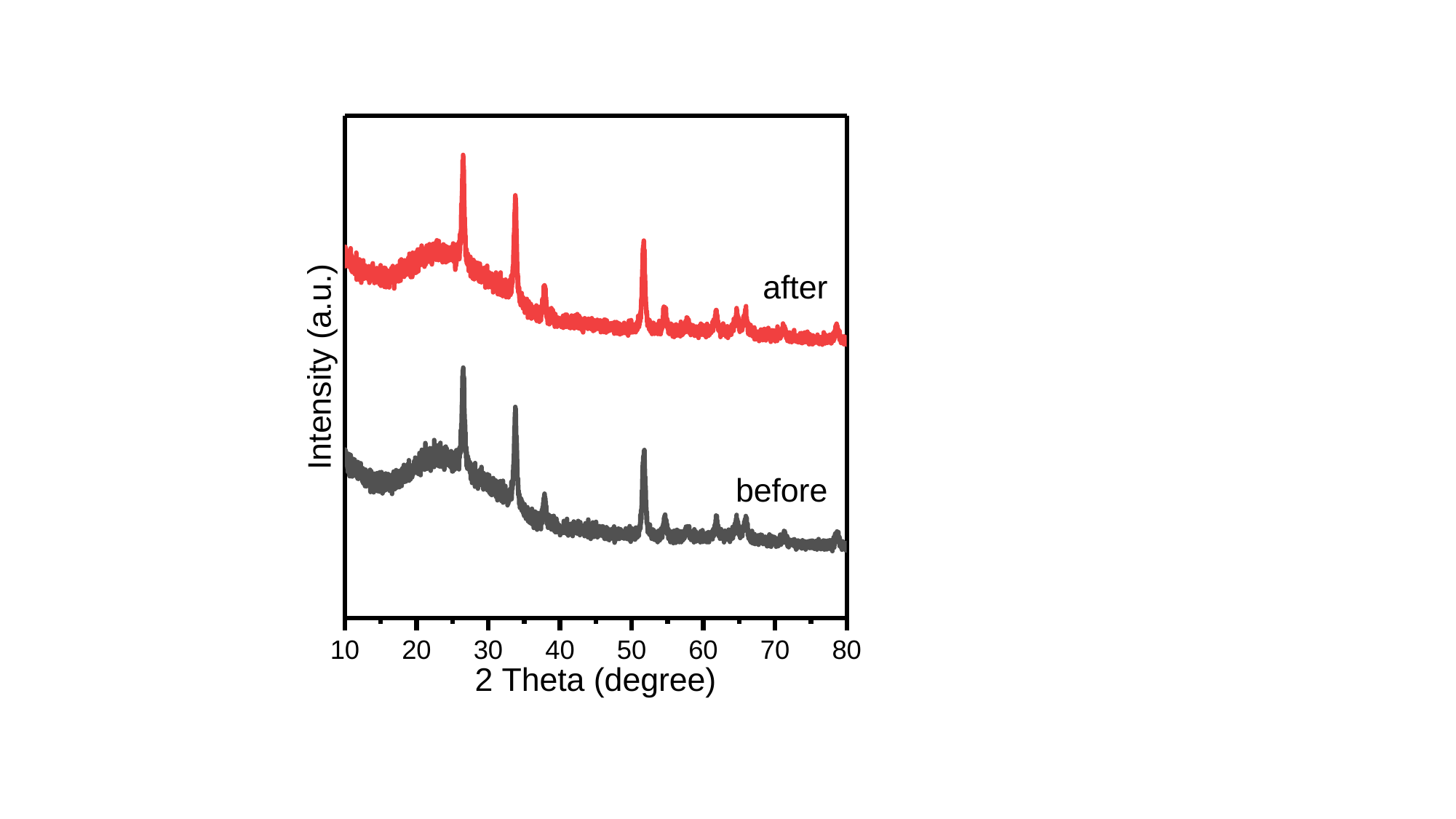

## Slide 21
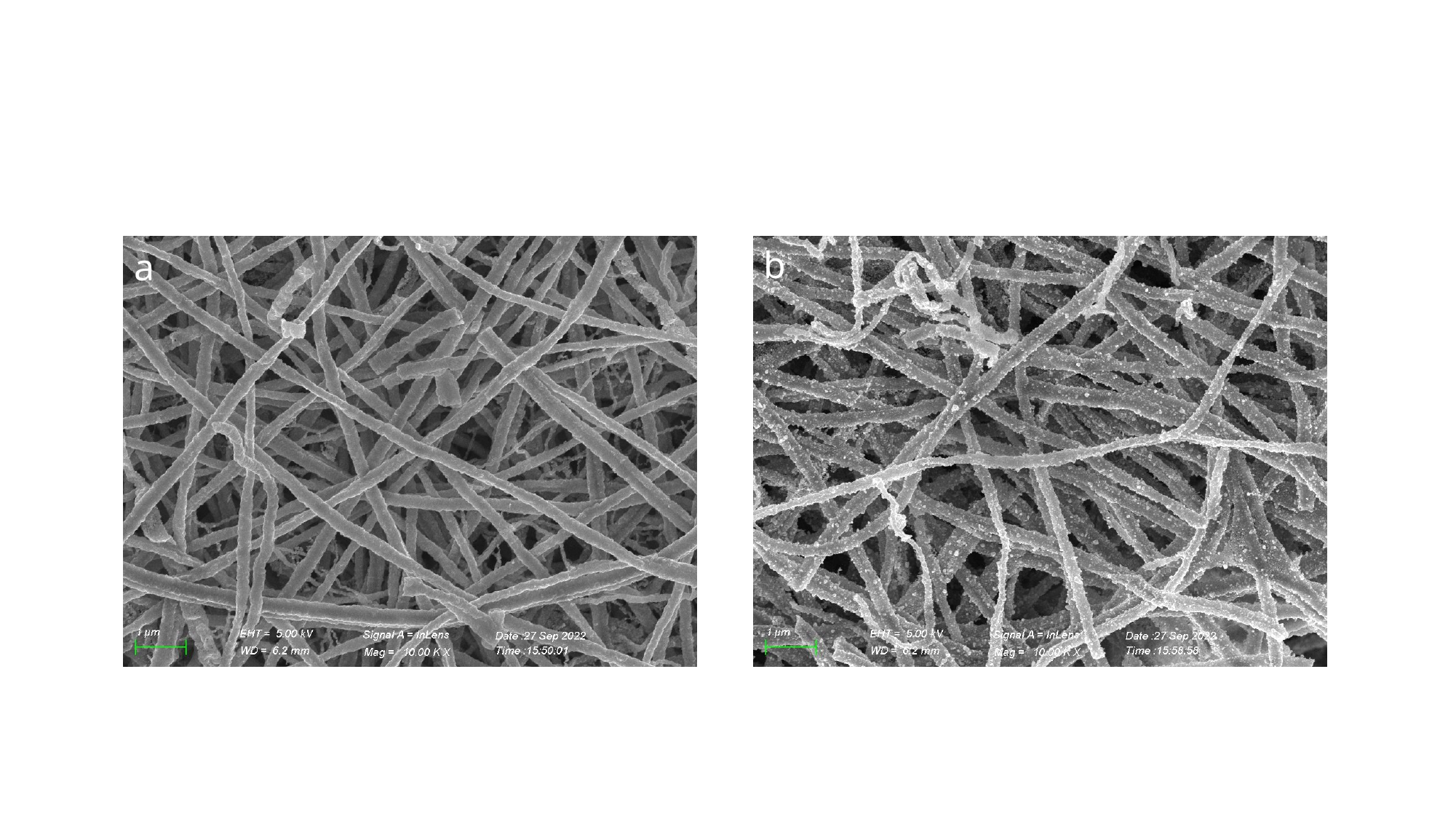

b
a

## Slide 22
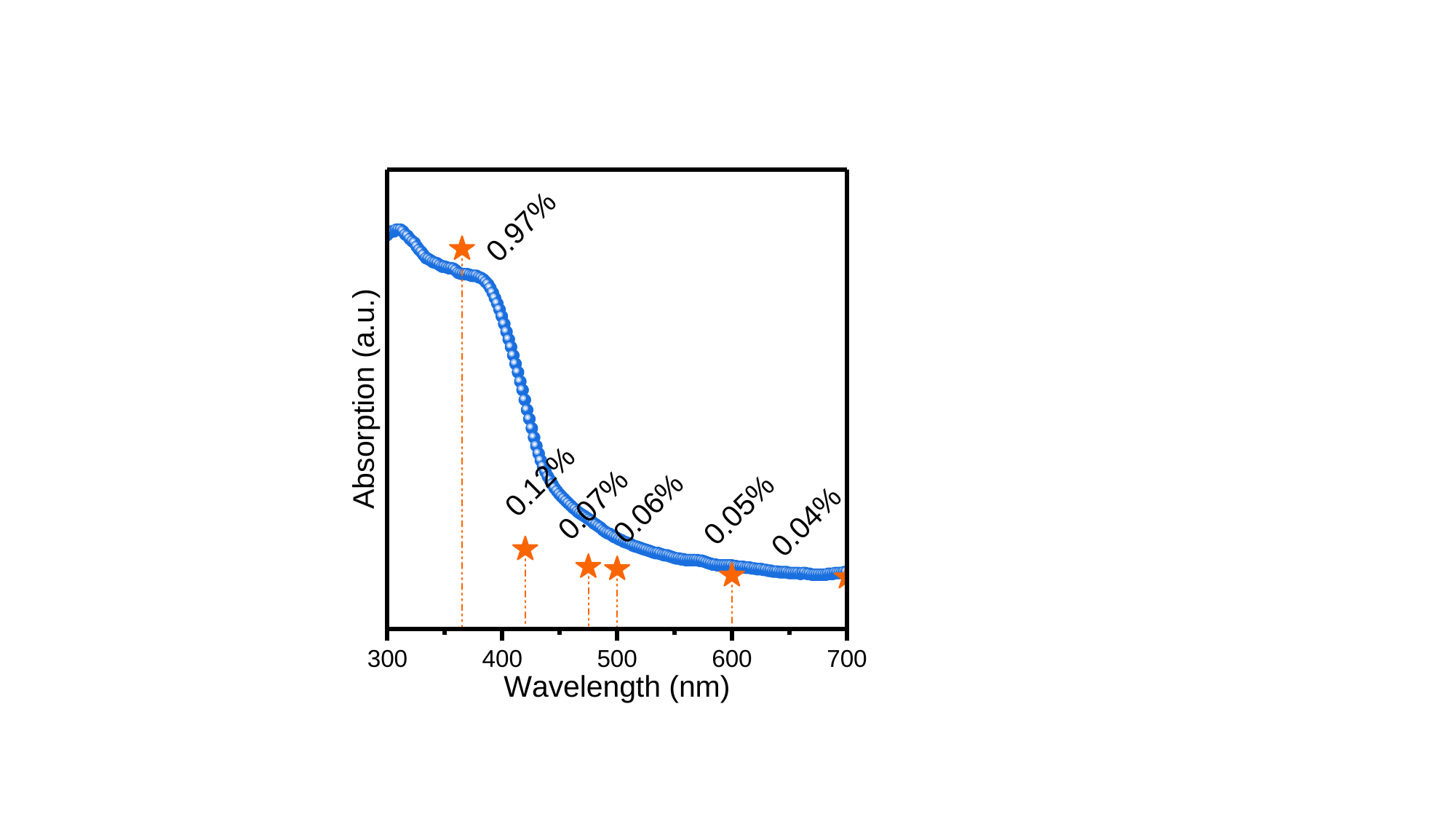

## Slide 23
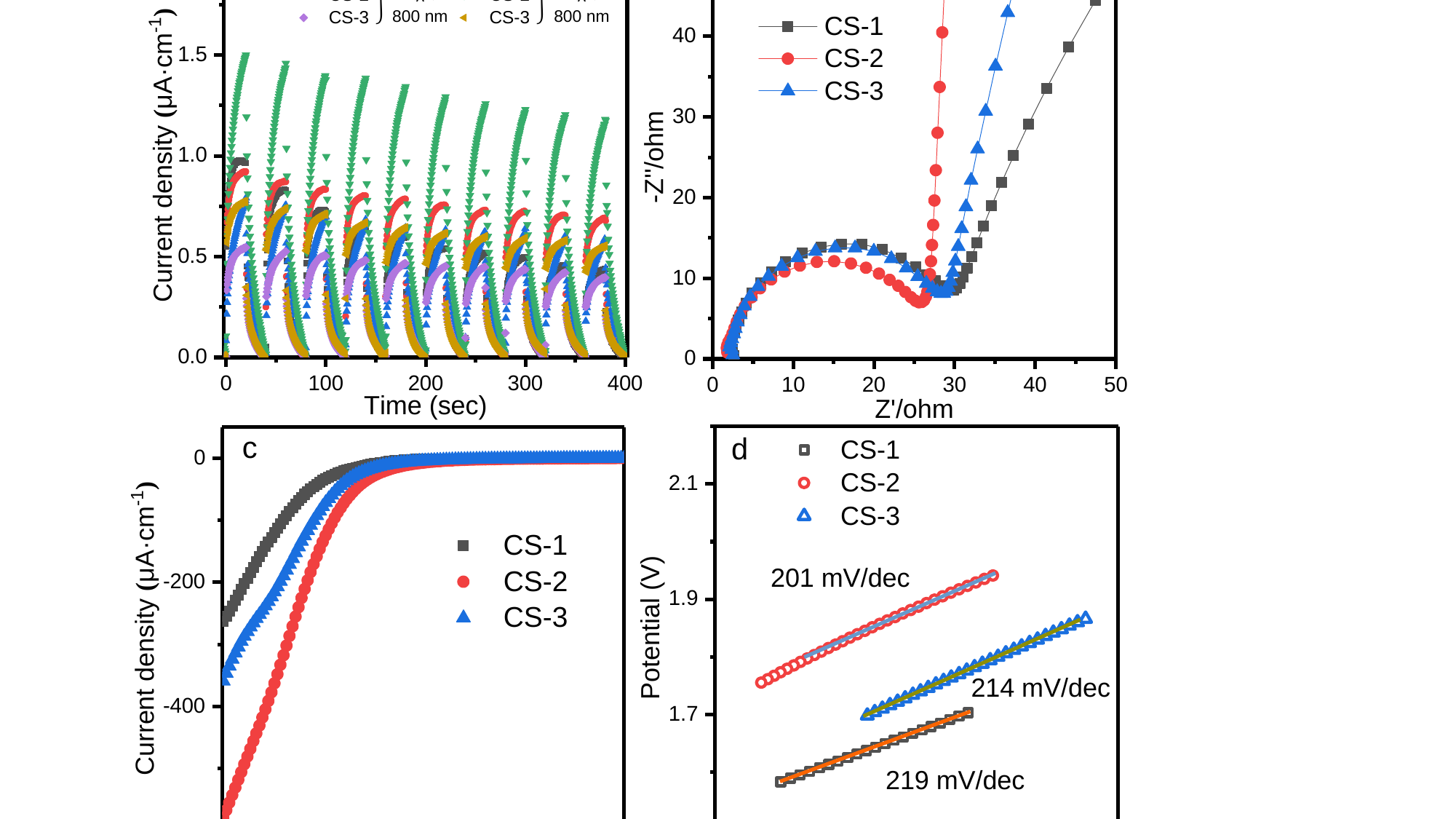

a
b
c
d

## Slide 24
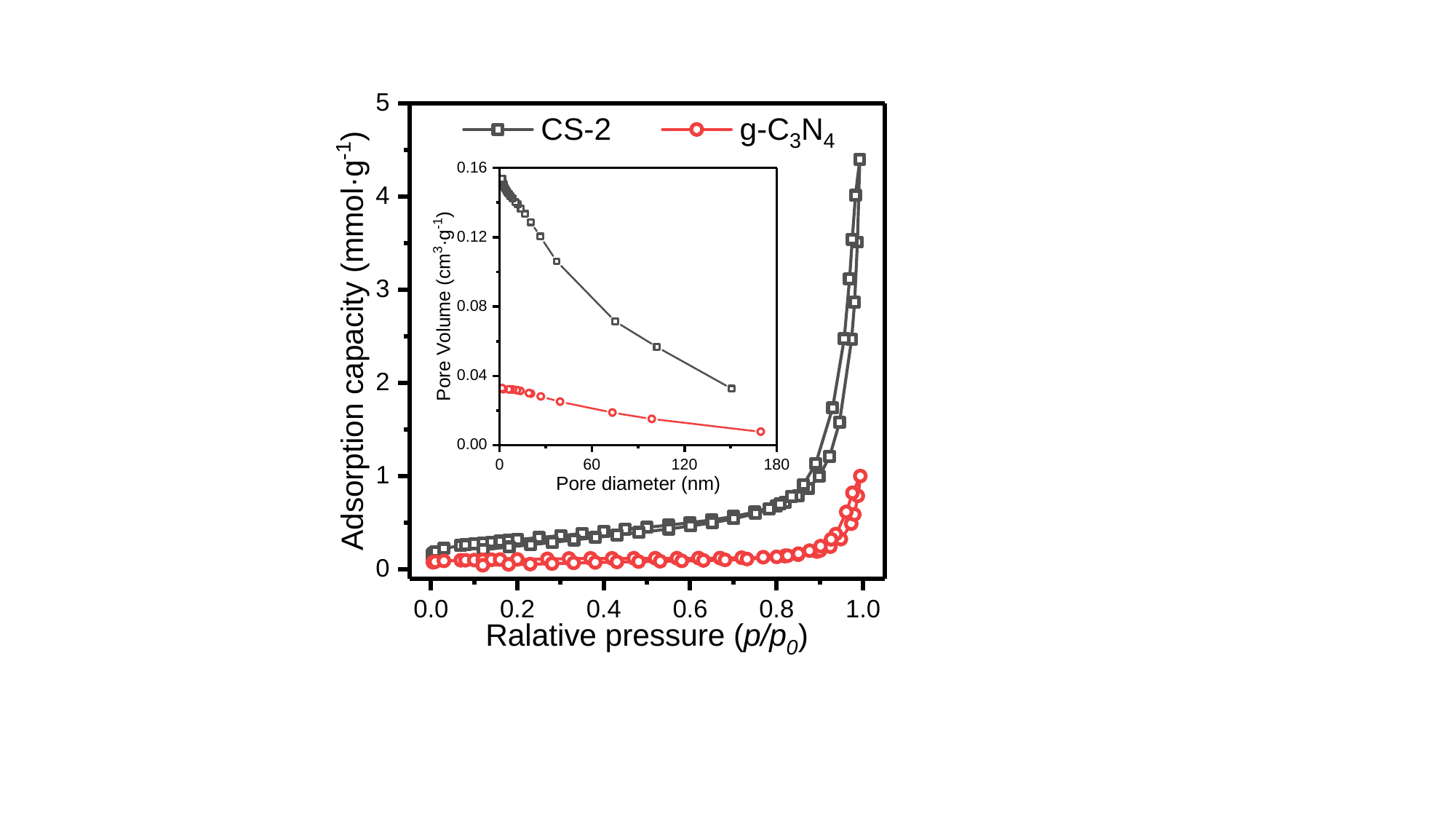

## Slide 25
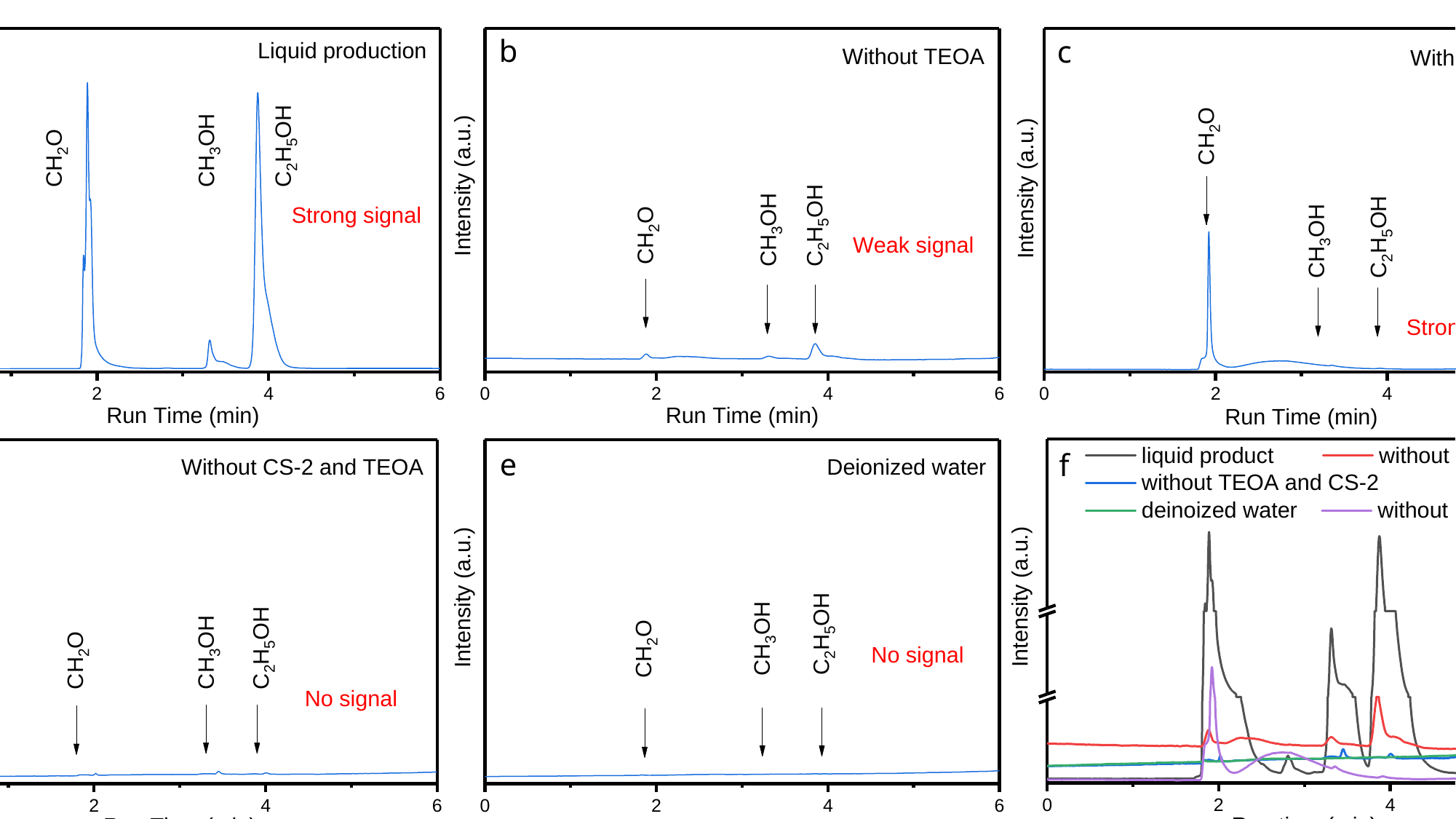

a
b
c
e
d
f
